# Oxoaporphine Metal Complexes (Co^II^, Ni^II^, Zn^II^) with High Antitumor Activity by Inducing Mitochondria-Mediated Apoptosis and S-phase Arrest in HepG2

**DOI:** 10.1038/srep46056

**Published:** 2017-04-24

**Authors:** Jiao-Lan Qin, Wen-Ying Shen, Zhen-Feng Chen, Li-Fang Zhao, Qi-Pin Qin, Yan-Cheng Yu, Hong Liang

**Affiliations:** 1 State Key Laboratory for Chemistry and Molecular Engineering of Medicinal Resources, School of Chemistry and Pharmacy, Guangxi Normal University, 15 Yucai Road, Guilin 541004, P. R. China

## Abstract

Three new oxoaporphine Co(II), Ni(II) and Zn(II) complexes **1**–**3** have been synthesized and fully characterized. **1**–**3** have similar mononuclear structures with the metal and ligand ratio of 1:2. **1**–**3** exhibited higher cytotoxicity than the **OD** ligand and cisplatin against HepG2, T-24, BEL-7404, MGC80–3 and SK-OV-3/DDP cells, with IC_50_ value of 0.23−4.31 μM. Interestingly, 0.5 μM **1**–**3** significantly caused HepG2 arrest at S-phase, which was associated with the up-regulation of p53, p21, p27, Chk1 and Chk2 proteins, and decrease in cyclin A, CDK2, Cdc25A, PCNA proteins. In addition, **1**–**3** induced HepG2 apoptosis via a caspase-dependent mitochondrion pathway as evidenced by p53 activation, ROS production, Bax up-regulation and Bcl-2 down-regulation, mitochondrial dysfunction, cytochrome *c* release, caspase activation and PARP cleavage. Furthermore, **3** inhibited tumor growth in HepG2 xenograft model, and displayed more safety profile *in vivo* than cisplatin.

While cisplatin and related platinum complexes have been successfully used as anticancer agents^1^,^2^. Developing non-platinum metal-based anticancer complexes has attracted the interests of many researchers. A large number of transition metal complexes with high antitumor activity have been reported^3^,^4^, including Co(II), Ni(II) and Zn(II). Some researches showed that cobalt(II) functioned as a metal center in cobalamines and vitamin B12 or as an ion stimulated oxidative stress through mitochondrial mediated apoptosis^5^. Nickel(II) complexes were shown as telomerase and topoisomerase II inhibitors^6^,^7^,^8^, inducing cancer cell apoptosis via mitochondrial pathways^9^,^10^. Increasing evidence indicated that zinc(II) complexes exhibited high anticancer activity, such as zinc(II) chelated dihalo-8-quinolinol^11^, isoquinoline derivatives^10^, phthalocyanine^12^, thiosemicarbazone^13^,^14^,^15^, and etc. In addition, Zn(II) complexes of some thiosemicarbazones possess intrinsic fluorescence, which enables the use for fluorescence imaging and further study of their mechanism of action^16^,^17^ Thus, Zn(II) complexes were shown to have the potential as novel anticancer agents.

Many efforts have been made on the development of new effective antitumor metal complexes via variation of coordination modes, metals or ligands, to trigger apoptosis. This is currently one of the most promising strategies to develop new DNA-targeting anticancer agents for chemotherapy. Recently, metal complexes based on traditional Chinese medicines (TCMs) provided a novel approach to potential (pro-) drugs^18^,^19^. Oxoaporphine and oxoisoaporphine alkaloids, isolated from a number of traditional Chinese medicines, were shown to possess antitumor activity^20^,^21^,^22^. The Pt(II), Ru(II), Au(III), Y(III) and Dy(III) complexes of oxoaporphine or oxoisoaporphine have been synthesized and their antitumor activities have also been studied^23^,^24^,^25^,^26^. Mechanistic studies revealed that these metal complexes targeted DNA and inhibited topoisomerase I, which effectively inhibited cell growth by inducing S-phase arrest and apoptosis via the mitochondrion-mediated pathway. Two oxoisoaporphine-Pt(II) complexes^25^ and a water-soluble oxoaporphine-Ru(II) complex were found as telomerase inhibitors targeting c-myc, telomere, and bcl-2 G4s, and triggering apoptosis and cell senescence^26^. All these studies indicate that oxoaporphine alkaloids are potential ligands for the development of metal-based antitumor agents.

In addition, Co(II), Ni(II) and Zn(II) complexes of oxoaporphine or oxoisoaporphine have also been synthesized and their antitumor activities have been studied, which were listed in Table 1^27^,^28^,^29^,^30^,^31^. However, the mechanism of cell death on the molecular level (such as necrosis and apoptotic regulation) of these Co(II), Ni(II) and Zn(II) complexes with new oxoaporphine alkaloids has not yet been fully explored. In order to achieve better application of oxoaporphine alkaloids, an oxoaporphine derivative (**OD**) and its Co(II), Ni(II) and Zn(II) complexes were synthesized. Their antitumor efficacy *in vitro* and *in vivo* was evaluated, and the antitumor mechanism was investigated.

## Results and Discussion

### Synthesis and Structural Characterization of OD and 1–3

**Figure 1:**
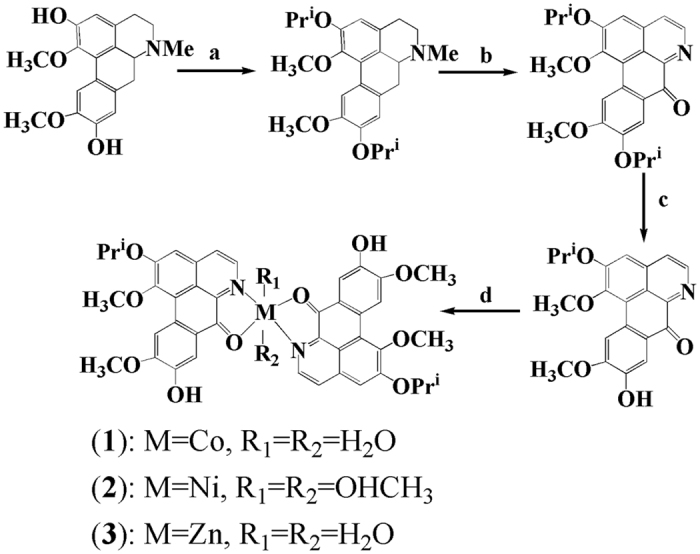
Synthetic routes for ligand (OD) and its metal complexes 1–3. The outsphere perchlorate anions have been omitted for clarity. Reagents are as follows: (**a**) (CH_3_)_2_CHI, anhydrous K_2_CO_3_ and ethanol (reflux, 12 h); (**b**) Pb(CH_3_COO)_4_, AcOH (75 °C, 8 h); (**c**) CH_3_COOH/H_2_SO_4_ (96:4) (N_2_, 75 °C, 20 h); (**d**) MeOH/CHCl_3_ (4:1) for **1** and **3**; MeOH/H_2_O (4:1) for **2**, (80 °C, 72 h).

The ligand **OD** was synthesized according to the previously reported methods (Fig. 1)^26^, in which (+)-boldine was used as the starting material. Complexes [Co(OD)_2_(H_2_O)_2_](ClO_4_)_2_ (**1**), [Ni(OD)_2_(CH_3_OH)_2_](ClO_4_)_2_ (**2**) and [Zn(OD)_2_(H_2_O)_2_] (ClO_4_)_2_ (**3**) were synthesized by reaction of OD with M(ClO_4_)_2_·6H_2_O (M = Co, Ni and Zn) in MeOH/CHCl_3_ (4:1) (for **1** and **3**), and in MeOH/H_2_O (4:1) (for **2**), respectively. The metal complexes were characterized by IR, ESI-MS and elemental analyses. Their crystal structures were determined by single-crystal X-ray diffraction analysis.

### Crystal Structures of 1–3

**Figure 2:**
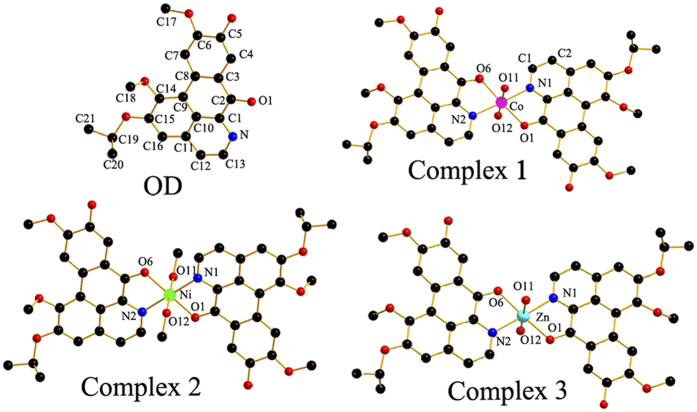
Crystal structures of OD and complexes 1–3. The outsphere perchlorate anions have been omitted for clarity.

The crystal data and refinement details of **1**–**3** and **OD** were summarized in Table S1 (Supporting Information), and selected bond lengths and angles were listed in Table S2. The crystal structures of **1**–**3** and **OD** were shown in Fig. 2. **OD** possesses a planar structure with N(1) and carbonyl O(5) as potential donors. Complexes **1**–**3** have similar mononuclear structures. The central metal ion adopts six-coordinate distorted-octahedral geometry and is surrounded by two N and O atoms from the chelating **OD** ligands, two O from H_2_O (For **2**, two O atoms from CH_3_OH).

### Solution Stability

The stability of **1**–**3** in Tris-HCl buffer solution (TBS, pH = 7.35, containing 1% DMSO) for 0, 12 and 48 h were primarily analyzed by means of UV-Vis spectroscopy, as shown in Figure S1. It can be found that the UV-Vis spectra character of **1**–**3** retained without new emerging absorption peaks and no obvious hypochromic effect, suggesting that **1**–**3** were stable in solution over 48 h. The stability of **3** was also monitored by HPLC using a mobile phase of methanol/H_2_O (v:v = 80/20), no obvious changes of HPLC chromatograms of **3** in TBS were observed in 0 and 24 h (Figure S2). Bases on all these results, it indicated that **1**–**3** were stable under the physiological conditions.

### *In Vitro* Cytotoxicity

The *in vitro* cytotoxicity of **OD**, **1**–**3** and the corresponding metal salts were determined by MTT assay towards a panel of human tumor cells including HepG2, T-24, BEL-7404, MGC80-3, SK-OV-3/DDP and normal HL-7702 cells. As the positive control, cisplatin was dissolved at a concentration of 1 mM in 0.154 M NaCl^32^. In the preliminary screening test, the cells were treated with the compounds at a concentration of 20 μM for 48 h, and the inhibition rates were listed in Table S3. At 20 μM, **1**–**3** showed higher cytotoxicity against all the tested tumor cells than **OD** and the corresponding metal salts (**1**–**3** showed 60~90%, **OD** 33~47%, metal salts 11~36%, respectively).

The cytotoxicity of **1**–**3** was further quantitatively evaluated by IC_50_ values. As listed in Table 2, **1**–**3** showed lower IC_50_ values against the most selected tumor cells than free ligand **OD** and cisplatin, especially to HepG2, T-24, and SK-OV-3/DDP cells (<0.7 μM). For example, **1**–**3** exhibited preferential cytotoxicity toward HepG2 (IC_50_ = 0.66 ± 0.11, 0.20 ± 0.09 and 0.39 ± 0.03 μM, respectively), which were approximately 14–45 fold increments comparing to the free **OD** ligand (IC_50_ = 8.98 ± 0.76 μM), 12–39 fold increments comparing to cisplatin (IC_50_ = 7.72 ± 0.83 μM). Complexes **1**–**3** showed almost the same cytotoxicity against T-24 tumor cells, with 18–40 times higher cytotoxicity than **OD**, 15–33 times higher cytotoxicity than cisplatin. It is worth mentioning that **OD** was not sensitive to SK-OV-3/DDP (IC_50_ = 38.53 ± 1.42 μM), only 2 folds higher than cisplatin (IC_50_ = 65.97 μM), while **1**–**3** exhibited IC_50_ values of 0.18–0.57 μM which was 116–366 folds higher than cisplatin. In addition, although **1**–**3** also exhibited toxicity in human normal live cell HL-7702, they showed higher cytotoxicity towards tumor cells HepG2, T-24 and SK-OV-3/DDP, suggesting that **1**–**3** were more selective to tumor cells. These observations suggest that **1**–**3** have different antitumor mechanism from that of cisplatin, and **1**–**3** have great potential as metal-based anticancer agents.

### Cell Morphological Examination by Hoechst33258 and AO/EB

**Figure 3:**
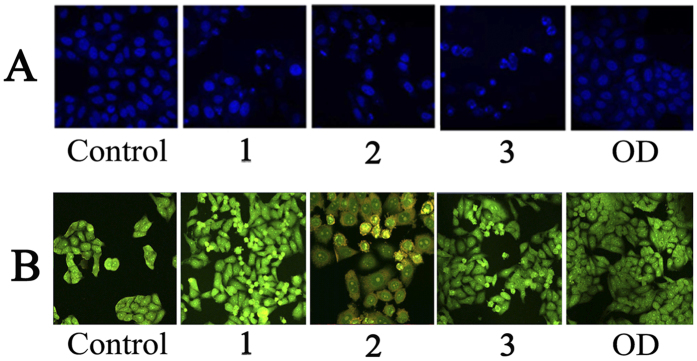
HepG2 cells morphological examination. (**A**) Hoechst-33258 staining was used for apoptotic nuclear morphological analysis after 24 h treatment of **1**–**3** and **OD** in HepG2 cells. Images were acquired using a Nikon Te2000 deconvolution microscope (magnification 200x). (**B**) AO/EB molecular probes detected apoptosis cells of HepG2 treated by 1.0 μM of **1**–**3** and **OD** for 24 h, respectively. Images were acquired using a Nikon Te2000 deconvolution microscope (magnification 200x).

Considering the high cytotoxicity of **1**–**3** and **OD** to HepG2 cells, we determined their effects on cell apoptosis using Hoechst33258 and AO/EB probes^33^, HepG2 tumor cells were incubated with **1**–**3** and **OD** for 24 h and stained with membrane-permeable blue Hoechst 33258 to detect apoptotic morphological changes by fluorescence microscope. As shown in Fig. 3A, it was found that the cell morphology of HepG2 cells in the control group remained regular with round contours. However, those treatments with 1.0 μM **1**–**3** showed significant characteristic apoptosis, such as irregular nuclei, chromatin shrinkage, and formation of apoptotic bodies. Under the same incubation (or treated) conditions, the AO/EB molecular probes were further used to detect apoptotic cells. The apparent morphological changes of the HepG2 cells were also observed when they were treated with **1**–**3** and **OD**. As shown in Fig. 3B, **1**–**3** caused a large number of HepG2 cells to shrink and undergo apoptosis, which suggested that **1**–**3** induced more cell apoptosis than **OD** ligand.

### Cell Cycle Analysis

**Figure 4:**
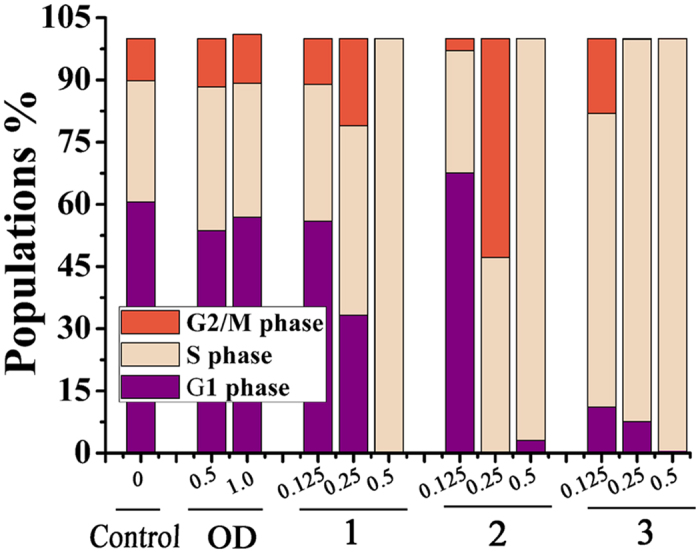
Effect OD and 1–3 on HepG2 cells cycle distribution. Cell cycle profiles were measured by flow cytometry after treating HepG2 cells with **1**–**3** (0.125, 0.25 and 0.5 μM) and **OD** (0.5 and 1.0 μM) for 24 h. Graph bars show the distributions of cells in different phases of cell cycle.

With the high cytotoxicity *in vitro*, HepG2 cells were selected to investigate the cell cycle treated with **1–3** (0.125, 0.25 and 0.5 μM) and **OD** (0.5 and 1.0 μM) for 24 h. As shown in Figs 4 and S3, **OD** barely change the population of the cell cycle compared to the untreated cells at 0.5 and 1.0 μM. However, **1–3** induced a significant increase of S-phase cell population in a dose-dependent manner. And surprisingly, 100% S-phase cells were observed after incubation with 0.5 μM **1–3**. All these results suggested that **1–3** exhibited inhibition of HepG2 cell growth which was associated with the cell cycle arrest in S-phase.

### Cell Cycle Related Proteins Expressions

**Figure 5:**
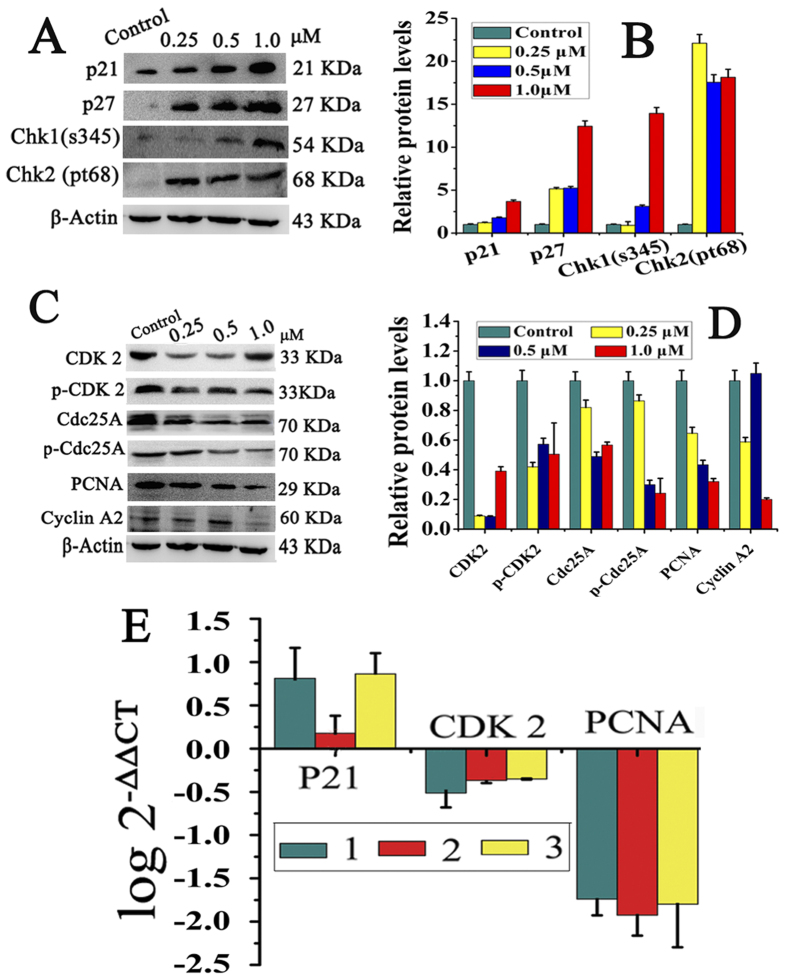
The levels of cell cycle regulatory proteins in HepG2 cells after treatment with **3**. (**A** and **C**) Cell cycle regulatory proteins analyzed by western blot under the same experiment condition after incubating HepG2 cells with **3** at 0.25, 0.5 and 1.0 μM for 24 h, respectively, where β-actin were used as the internal reference. (**B** and **D**) The relative protein expression of each band = (density of each band/density of β-Actin band). Mean ± SD was from three independent measurements. (**E**) RT-PCR to determine the expression of p21, CDK2 and PCNA in the HepG2 cells. Cells were treated with **3** at 1.0 μM for 24 h.

Chk1 and Chk2 are the most important signal transducers for cell cycle regulation and DNA damage checkpoint responses^34^. In order to explore the mechanism of **1–3** induced S-phase arrest, the cell cycle-related proteins were evaluated by Western blotting and RT-PCR assay. Because **1–3** have similar structures, similar effects on cell cycle-regulatory proteins were observed. Taken **3** as an example (Fig. 5), significant increase in the expression levels of Chk1 and Chk2 were observed in **3**-treated HepG2 cells, which indicated that **3** induced DNA damage and the S-phase checkpoints were activated^35^,^36^. It also caused a concentration-dependent increase in the expression of CDKs inhibitor proteins p21 and p27, comparing to the control group. Obviously, when increasing p21 and p27 protein levels, the levels of cyclin A2 and CDK2 were down regulated, so did PCNA and Cdc25A. It was reported that upregulation of p21 in human cancer cells could inhibit the expression of cell cycle regulatory proteins such as cyclin A and CDK2^33^. Similarly, p-CDK2, and p-Cdc25A showed a significant decrease when treated with **3**. The same effects on the cell cycle regulatory proteins were also observed with **1** and **2**. As shown in Figures S4 and S5, the levels of Chk1, Chk2, p21 and p27 increased dramatically, while the levels of cyclin A, CDK2, PCNA, Cdc25A decreased significantly. The upregulation of p21 and p27 but downregulation of cyclin A, CDK2 and CDK1, suggested that **1–3** mediated HepG2 cells arrested in S phase^37^. P21 can also inhibit DNA synthesis directly by binding to PCNA^38^, which further confirmed S-phase arrest.

### Comet Assay

**Figure 6:**
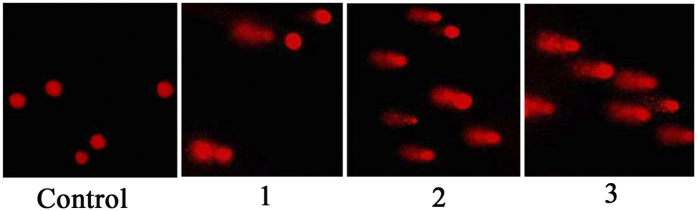
1–3 induced DNA damage in HepG2 cells. Cells were treated with **1**–**3** (1.0 μM) for 24 h. The length of the tail reflects the DNA damage in cells.

After the observation of significant S-phase arrest caused by **1–3**, the study of DNA damage assay is necessary. The single cell gel electrophoresis technique (comet assay) is a simple, sensitive, reliable, and rapid method to detect DNA damage in eukaryotic cell^39^. As shown in Table 3 and Fig. 6, **1–3** at 1.0 μM induced DNA damage (fragmentation or unwinding) appeared as fluorescent comets with remarkable increase of tails size, especially for **3**. However, in the control, the normal undamaged DNA does not migrate far away from the origin position. These results demonstrated that **1–3** inhibited HepG2 cell growth via the induction of DNA damage and associated cell cycle arrest in S phase^40^.

### **1–3** Mediated S-Phase Arrest via ATM-Chk2-Cdc25A-Cdk2 and ATR-Chk1-Cdc25A-Cdk2 Pathways

As known, the protein kinases ATM (ataxia-telangiectasia, mutated) and ATR (ATM and Rad3-related) are potential sensors of DNA damage, which conduct DNA damage signaling to downstream effectors Chk1 and Chk2^41^,^42^. As discussed above, **1–3** caused remarkable DNA damage, and activated signaling Chk1/2 kinases. Then the DNA damage signaling translated to the downstream effector proteins, Cdc25A, cyclin-dependent kinases (cyclin A2) and CDK2, which culminated in the S-phase arrest of cell cycle progression^37^,^39^. All these results demonstrated that **1**–**3** caused S-phase arrest via the activation of ATM-Chk2-Cdc25A-Cdk2 and ATR-Chk1-Cdc25A-Cdk2 pathways, very similar to other oxoaporphine or oxoisoaporphine alkaloid-metal complexes^23^,^25^,^26^.

### Cell Apoptosis Assay by Flow Cytometry

**Figure 7:**
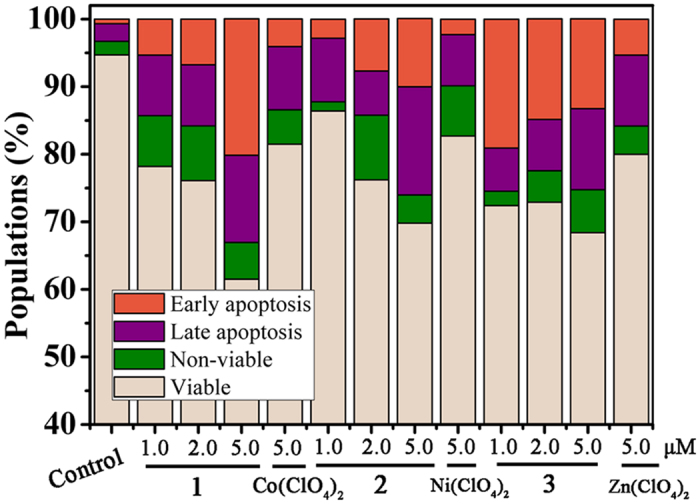
Effects of OD, complexes 1–3 and the corresponding metal salts on cell apoptosis. Cell apoptosis was analyzed by flow cytometry and Annexin V-FITC/PI double staining after incubating HepG2 cells with **OD**, complexes **1**–**3** and the corresponding metal salts at different concentrations for 24 h.

The quantitative analysis of apoptotic HepG2 induced by **1–3** (1.0, 2.0 and 5.0 μM) and their corresponding metal salts (5.0 μM) after 24 h treatment, were investigated by flow cytometry and Annexin V-FITC/PI double staining. As depicted in Figs 7 and S6, **1–3** caused a significant increase in apoptotic cells comparing to the untreated cells. After treating cells with 5.0 μM **1–3**, the population of apoptotic cells were 33.1%, 26.1% and 25.3%, respectively, suggesting that **1–3** could cause apoptosis.

### Detection of Apoptosis Proteins by Western Blotting

**Figure 8:**
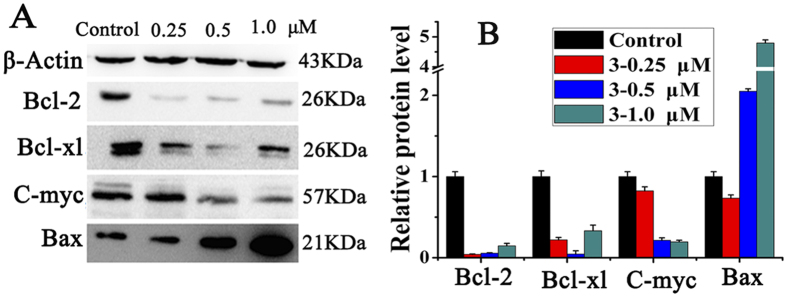
Effect of 3 on the apoptosis protein levels in HepG2 cells. (**A**) Western blot analysis of Bcl-2, Bcl-xl, C-myc and Bax after treatment of HepG2 cells with **3** at 0.25, 0.5, 1.0 μM, respectively for 24 h. The apoptotic proteins were measured under the same experiment condition, and β-actin was used as the internal reference. (**B**) Densitometry analysis of Bcl-2, Bcl-xl, C-myc and Bax band from part A. The relative expression of each band = (density of each band/density of β-actin band). Mean and SD values were from three independent measurements.

To further investigate the apoptotic pathway activated by **1–3**, the expression levels of a series of anti-apoptotic and pro-apoptotic proteins in inducing cell death and cell apoptosis were analyzed. It is well known that Bcl-2 family proteins are key regulators of mitochondrial membrane potential and are strongly associated with apoptotic cell death^43^. As shown in Fig. 8, the expression levels of proteins Bcl-2, Bcl-xl and c-Myc were remarkably down-regulated after incubating with **3**, while the expression of pro-apoptotic proteins Bax was significantly increased, which were proved by multiple assays and exposures (Figure S7). As expected, similar results were observed when treating with **1** and **2** under the same experiment conditions, that the expressions levels of Bcl-2, Bcl-xl and c-myc proteins were down-regulated (Figure S8).

### Induced Ca^2+^ Fluctuation

**Figure 9:**
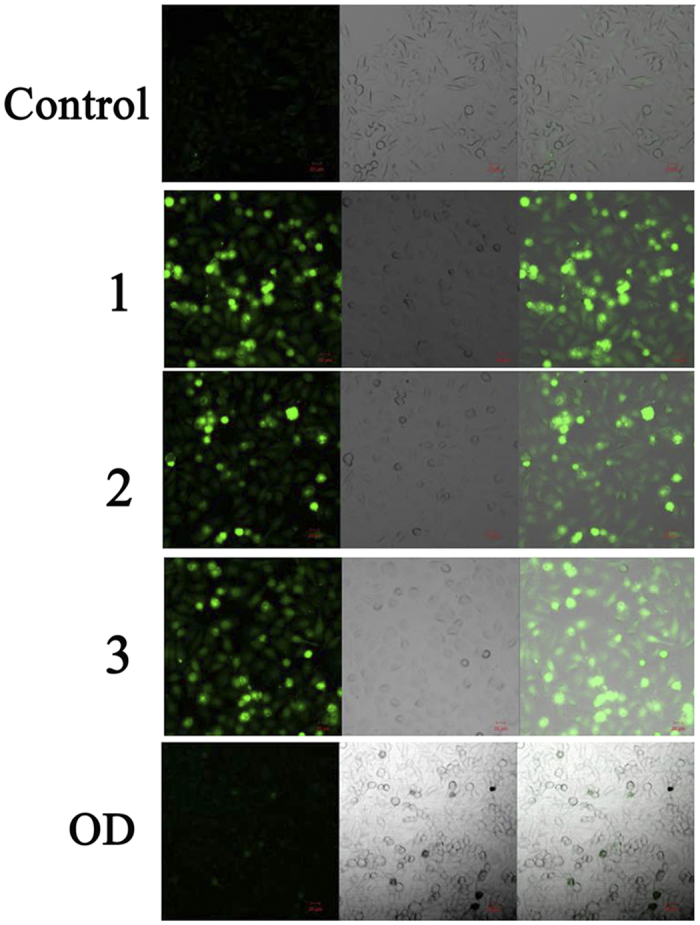
1–3 and OD affected the levels of intracellular Ca^2+^. Fluo-3AM was used, and green fluorescence demonstrated the increased of Ca^2+^.

It was reported that the increase of intracellular free Ca^2+^ may disrupt the mitochondrial membrane potential and ultimately lead to cell apoptosis and death^44^. Thus, the fluorescent probe Fluo-3AM is used to detect the intracellular calcium level. When it combines with Ca^2+^, its fluorescence increases about 80 times comparing to the free ligand. The intracellular Ca^2+^ level caused by treatment of **1–3** in HepG2 cells were shown in Fig. 9. In the control, no green fluorescence was observed, suggesting that the level of intracellular free Ca^2+^ was very low. When exposed to **1–3** at 1.0 μM for 24 h, the green fluorescence increased, indicating that the level of intracellular Ca^2+^ promoted by **1–3** increased steadily. The high level of intracellular Ca^2+^ and decrement of MMP (Δψm) may lead to the release of cytochrome c from mitochondria^45^.

### Assay of Reactive Oxygen Species (ROS) Generation

**Figure 10:**
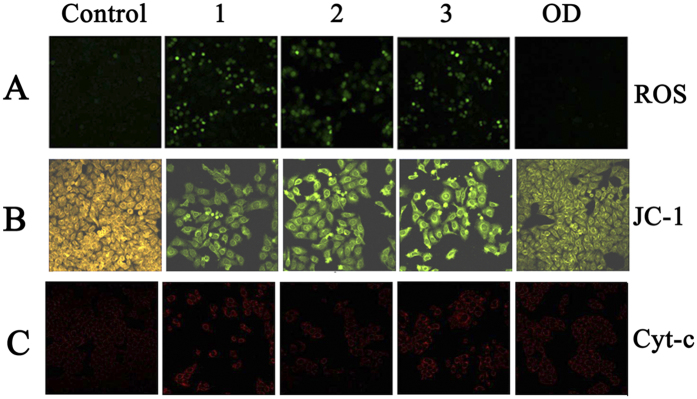
Fluorescence microscope analysis of the levels of ROS, ΔΨm and Cyt-*c* in HepG2 cells treated with 1–3. (**A**) ROS generation images under a fluorescence microscope after treatment with **1**–**3** and **OD** at 1.0 μM for 24 h. (**B**) JC-1 staining images after HepG2 cells treatment with **1**–**3** and **OD** at 1.0 μM for 24 h. Selected fields illustrated the corresponding live cells (orange-red), apoptotic cells (green), and images were acquired using a Nikon Te2000 microscope. (**C**) Cyt-*c* images under a fluorescence microscope after treatment with **1**–**3** and **OD** at 1.0 μM for 24 h. (magnification 200x).

In general, antitumor active compounds lead to the generation of ROS (reactive oxygen species)^46^. Overproduction of ROS may cause oxidative damage of DNA, mitochondrial dysfunction and even result in cell apoptosis^47^. As shown in Fig. 10A, comparing to the control, the intensity of green fluorescence increased significantly after treatment with **1–3** at 1.0 μM, whereas the fluorescence was barely observed when cells were treated with **OD,** which indicated that **1–3** greatly raised the released ROS level in the HepG2 than **OD** did. The overproduction of ROS induced by **1–3** might lead to direct DNA breakage, mitochondrial dysfunction, and apoptosis in HepG2 cells^46^.

### The Mitochondrial Membrane Potential (MMP) (ΔΨm)

The loss of mitochondrial membrane potential (MMP, Δψ_m_) is generally considered as an early event in the apoptotic process^48^. The changes of MMP in the HepG2 cells caused by **1–3** and **OD** are detected by the fluorescent probe, JC-1, which emits orange-red fluorescence in healthy cells, and then remains as a green fluorescent monomer due to the loss of Δψ^49^. As shown in Fig. 10B, the control cells emitted orange fluorescence suggesting the coupled mitochondria with a normal Δψ. However, notable green fluorescence has been observed when the cells were incubated with **1–3** and **OD** at1.0 μM, indicating that **1–3** and **OD** promoted the loss of Δψ, and the effect of **OD** was relatively weaker than that of **1–3** at the same concentrations. The results indicated that **1–3** induced the reduction of MMP, which facilitated the apoptosis of HepG2 cells^50^.

### Release of Cytochrome *c*

**Figure 11:**
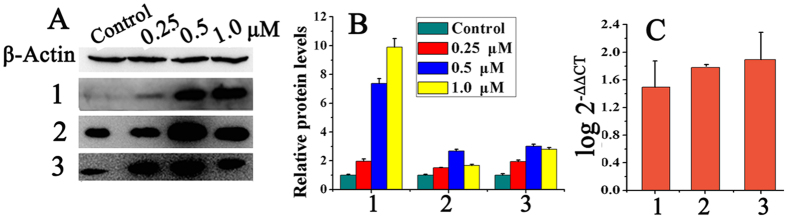
Effects of 1−3 on the protein expression level of cytochrome *c* in HepG2 cells. (**A**) Western blot analysis of cytochrome *c* after treatment of HepG2 cells with **1–3** at 0.25, 0.5, 1.0 μM for 24 h under the same experiment condition, respectively. (**B**) Densitometry analysis of cytochrome *c* band from part A. The relative expression of each band = (density of each band/density of actin band). Mean and SD values were obtained from three independent measurements. (**C**) RT-PCR to determine the expression of cytochrome *c* in the HepG2 cells treated with **1–3**.

Cytochrome *c* (Cyt-*c*) is released from mitochondrial membrane space due to the mitochondrial dysfunction, which can induce cell apoptosis by activating caspases proteases^51^. **1–3** can cause great decrease of mitochondrial membrane potential (Δψ_m_), so it is necessary to detect the levels of cytochrome *c* in HepG2 cells. After incubated with Cy3, the intensity of red fluorescence was greatly enhanced in HepG2 cells which were treated with **1–3** and **OD** (Fig. 10C), especially with the metal complexes, indicating that the intracellular levels of cytochrome *c* increased. Quantitative analysis were further confirmed by Western blot (Fig. 11A and B), compared with the control cells, the expression levels of cytochrome *c* increased significantly in a concentration-dependent manner after incubating HepG2 cells with **1–3** at 0.25, 0.5 and 1.0 μM, respectively. RT-PCR analysis also showed the up-regulation of cytochrome *c* (Fig. 11C).

### Caspases Activation by **1–3**

**Figure 12:**
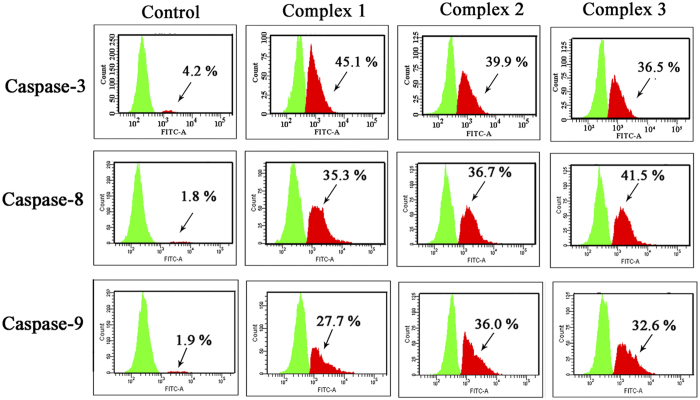
Effects of 1–3 on the expression levels of caspase-3/-8/-9 proteins. Caspase-3/-8/-9 were assessed by using the CasPGLOW^TM^ fluorescein activity caspase-3/-8/-9 staining kit by flow cytometry after treatment of HepG2 cells with complexes **1**–**3** at 1.0 μM for 24 h.

Caspase family is closely related to the cell apoptosis *via* the mitochondrial pathway^52^, especially caspases-3, -8 and -9. To better understand the antitumor mechanisms, it needs to confirm whether caspase-3, -8 and -9 are activated when the tumor cells are treated with **1–3**. As shown in Fig. 12, HepG2 cells were treated with 1.0 μM **1–3** for 24 h, additional strong peaks of the activated caspase-3, -8 and -9 were observed and the population of activated caspase increased from a range of 1.8–4.2% to 7.7–45.1%. Compared with the control, the proportions of the activated caspase-3 induced by **1**–**3** increased from 4.2% to 45.1%, 39.9% and 36.9%, respectively; the activated caspase-8 increased from 1.8% to 35.3%, 36.7% and 41.5%, respectively; and the activated caspase-9 increased from 1.9% to 27.7%, 36.0% and 32.6%, respectively. The up-regulation of the caspase suggested that **1–3** were efficient activators of caspase-3, -8 and -9, which caused apoptosis in HepG2 cells^53^.

### Induce Apoptosis via Mitochondrial Pathway and Fas-associated Signaling

**Figure 13:**
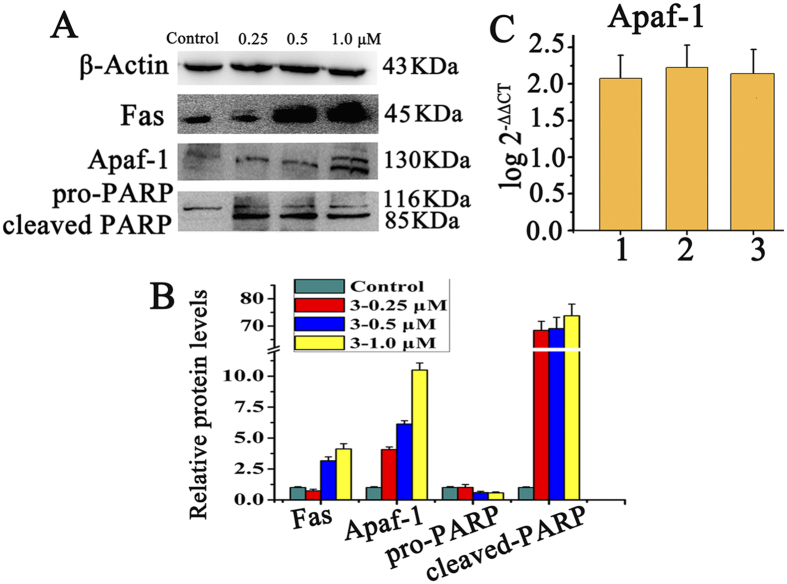
Effects of 1–3 on the expression level of apoptotic proteins in HepG2. (**A**) Western blot analysis of Apaf-1, PARP and Fas proteins in HepG2 cells with **3** at 1.0 μM for 24 h under the same experiment condition. (**B**) The relative expression of proteins from A, each band = (density of each band/density of β-actin band). Mean ± SD was obtained from three independent measurements. (**C**) RT-PCR to determine the expression of Apaf-1 in the HepG2 cells treated with **1**–**3**.

During the caspases-activated apoptotic cascades, mitochondrial apoptotic genes Apaf-1, PARP play an important role. The expressions of Apaf-1, pro-PARP and cleaved PARP in HepG2 cells treated with **3** were carried out by Western blotting. As shown in Fig. 13, the expression levels of Apaf-1, cleaved PARP increased significantly while pro-PARP decreased after incubating HepG2 cells with **3** at 0.25, 0.5 and 1.0 μM. The results indicated that complex **3** up-regulated these proteins in a concentration-dependent manner. The expression of Apaf-1 protein was further confirmed by RT-PCR (Fig. 13C) that the up-regulation of Apaf-1 was also observed. PARP was considered to be an important indicator of cell apoptosis, and the cleaved PARP could cause cellular inactivation and prevent DNA repair^54^. It is noteworthy that, approximately 70-fold increments of cleaved PARP was observed after HepG2 cells was incubated with **3**, multiple assays and exposures proved the result (Figure S9), indicating that **3** induced more cell apoptosis^37^.

In this assay, significant increases of the expression level of Fas cell surface death receptors was also found after treatment of HepG2 cells with **3** at 0.25, 0.5 and 1.0 μM for 24 h. Combined with the results above, **3** led to a great increase of caspase-8 and caspase-3, which indicated that besides the mitochondria intrinsic pathway, **3** could cause cell death extrinsically via the Fas-associated pathway^33^,^47^. Therefore, the antiproliferative activity of **1–3** is believed to occur via both mitochondria-mediated and death receptor-mediated apoptosis pathway.

### Complexes **1–3** down-regulated Topoisomerase II and up-regulated p53 in HepG2 cells

**Figure 14:**
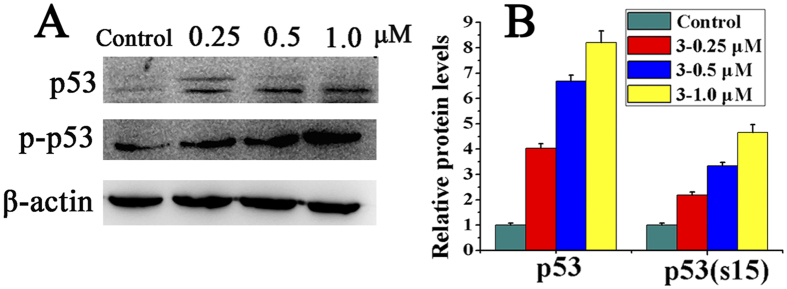
Effect of complex 3 on p53 and p-p53 expression. (**A**) Western blot analysis of p53 and p-p53 proteins in HepG2 cells with **3** at 0.25, 0.5 and 1.0 μM for 24 h. (**B**) The relative expression of proteins from A, each band = (density of each band/density of β-actin band). Mean ± SD was obtained from three independent measurements.

In response to oxidative stress, p53 becomes activated and the activation is marked by a quick accumulation of p53 in the stressed cells^55^, followed by phosphorylation^56^. In addition, topoisomerase II is at its maximum level in the late S phase and the G2/M phase^57^,^58^. Therefore, the effects of HepG2 on the topoisomerase II, p53 and p-p53 protein expression in p53 wild-type HepG2 cells were investigated by Western blot analysis. Complexes **1–3**, especially **3**, significantly decreased the level of Topoisomerase II expression in HepG2 cells (Figure S10). On the other hand, there was a concentration-dependent up-regulation of p53 and p-p53 expression in p53 wild-type HepG2 cells after induction with complexes **1–3** (Figs 14 and S11). Therefore, treatment with a high concentration of complexes **1–3** (0.5 μM) did not affect G2/M phase arrest, because at this concentration, the cell undergoes apoptosis before reaching to the G2 phase checkpoint. Indeed, these cells may follow another pathway that involves p53 at high concentrations rather than G2/M phase arrest, and thus can induce S-phase arrest in response to the replication blockade^59^. Therefore, the induction of wild-type p53 may also inhibit topoisomerase II gene expression, which could prevent the production of topoisomerase II at the level required to progress from S phase to G2/M phase.

**Figure 15:**
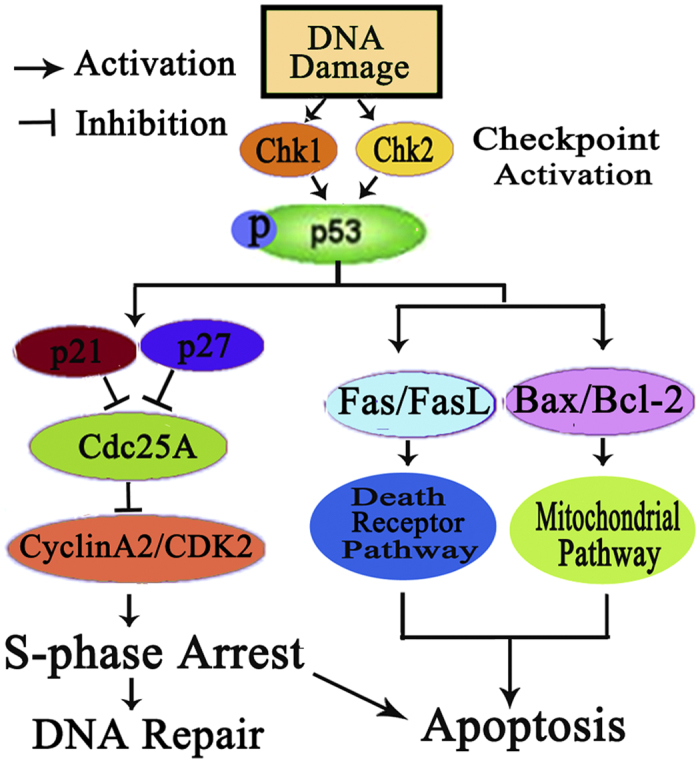
Proposed mechanisms of **1**–**3** induced p53-dependent DNA damage checkpoint activation, S-phase cycle arrest and apoptosis in HepG2 cells.

In summary, the results of this study demonstrate that three new complexes **1–3** with oxoaporphine derivative (**OD**), are active against all the selected cancer cell lines, and with higher activity in HepG2 cells. Complexes **1–3** significantly increased p53 and decreased topoisomerase II protein in HepG2 cells. Importantly, the antitumour effects of complexes **1–3** in HepG2 cells involve an interaction with topoisomerase II and the activation of p53-mediated mitochondrial apoptotic pathway. On the other hand, p53 plays as a key molecule in controlling cell responses to DNA damage through cell cycle arrest and apoptosis^60^, as shown in Fig. 15. Once p53 is activated, it holds the cell arrest in S phase by activating the expression of p21 and p27, and down-regulates Cdc25A and cyclin A2/CDK2. Thus, DNA repairs at this stage (S phase) or cell initiates apoptosis if DNA damage is shown irreparable^33^,^61^. On the other hand, activated p53 may initiate apoptosis and terminate cell proliferation through mitochondrial pathway by up-regulating the ratio of Bax/Bcl-2, causing mitochondrial dysfunction, activating caspase cascade, and ultimately leading to cell apoptosis (Fig. 15)^62^,^63^,^64^. In addition, activated p53 may also control apoptosis via death receptor pathway by activating Fas/FasL protein as reported^33^. All the results demonstrate that p53 plays as a central molecule in the cross-talk of cycle arrest and apoptotic signaling.

### Inhibited Tumor Growth in HepG2 Xenograft Nude Mice Model

**Figure 16:**
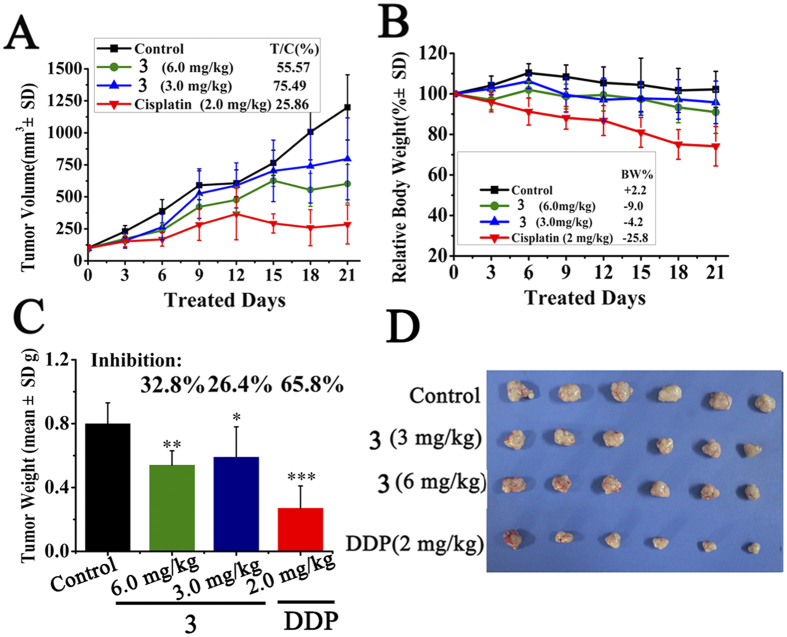
The *in vivo* antitumor activity of 3 in HepG2 xenograft model. (**A**) Tumor volume growth trend of control group, **3**-treated groups and cispaltin group. Tumor volumes were tracked by the mean tumor volume (mm^3^) ±SD (n = 6) and calculated as relative tumor growth rate [T/C%] values. (**B**) Tracking of body weight in mice during treatment. Relative body weight by considering the body weight on day 0 as 100%, the percent weight loss or gain was calculated on day 21. (**C**) Tumor growth inhibition rate. ^(*)^P < 0.05, ^(**)^P < 0.01, ^(***)^P < 0.001, p vs vehicle control. (**D**) Photographs of tumor from treatment groups and vehicle group.

To further evaluate the *in vivo* antitumor efficacy of these complexes, **3** was selected to inhibit tumor growth in a HepG2 xenografts model. The mice were randomized into control group, **3**-treated groups and positive control group (n = 6). Once the tumor had grown to an average volume of about 100 mm^3^, two group of nude mice were injected intraperitoneally (ip) of **3** at a high dose (6.0 mg/kg) and a low (3.0 mg/kg) dose daily for 20 days, respectively. Cisplatin was used as positive control injected at 2.0 mg/kg every two days, while control group received solvents only. As showed in Fig. 16, 3 can inhibit tumor growth significantly in a dose-dependent manner. After 20 days’ treatment, 6.0 mg/kg of **3** controlled tumor growth in volume with T/C 55.57% (p < 0.001), tumor weight inhibition rate with IR 32.8% (p < 0.05); meanwhile 3.0 mg/kg of **3** displayed with T/C 75.49% (p < 0.05), with IR 26.4% (p < 0.05), respectively. Cisplatin possessed higher inhibition activity to HepG2 xenograft model (T/C, 25.8%; IR, 65.8%, p < 0.001). However, a sharp drop in body weight (25.8%) was observed, more than permissible value (20%)^65^. In contrast, *in vivo* toxic effect of **3** was not obviously observed in nude mice since there was no significant loss in body weight among the solvent control group and **3**-treated groups. The results confirmed that **3** resulted in significant reduction in both tumor volume and weight of HepG2 xenograft *in vivo*. The relatively lower antitumor activity *in vivo* than *in vitro*, might be due to the poor solubility and low bioavailability of **3**, which indicated that further modification can be conducted to improve the solubility and bioavailability of these metal complex.

## Conclusions

Three new Co(II), Ni(II) and Zn(II) complexes of oxoaporphine were synthesized and fully characterized, and their *in vitro* and *in vivo* antitumor activities were evaluated. **1**–**3** possess higher cytotoxicity against five selected human tumor cells than the free ligand, especially the HepG2, T-24 and SK-OV-3 cells. **1–3** significantly induced S-phase arrest in HepG2 cells at low concentration, with the increase of Chk1, Chk2, p21, p27 and p53 expression and the decrease of cyclin A and Cdk2. In addition, **1–3** induced apoptosis in HepG2 cell via a caspase-dependent mitochondrial pathway and a Fas-associated extrinsic pathway. **1–3** induced activation of p53, production of ROS, increase of intracellular Ca^2+^, followed by promotion of Δψ loss, and ratio increase of Bax/Bcl-2, cytochrome *c*, and apaf-1. Then it cleaved caspase-3/9 and PARP, and finally caused HepG2 cell apoptosis. **3** also inhibited tumor growth of mice HepG2 bearing a xenograft model, and showed better safety profile *in vivo*. In conclusion, our results demonstrated the potential of **1–3** as the novel metal-based anticancer agents.

## Experimental Section

### Materials and instrumentation

All the chemical reagents used in the experiments were of analytical grade and commercially available. Ethidium Bromide (EB) and AO were purchased from Solar Technologies, Inc. MTT assay kit, propidium iodide (PI), antibody of Bax, Bcl-2, c-myc, CDK2, Cdc25A, Chk1/Chk2, caspase -3/-8/-9, PCNA and Fas were purchased from Abcam (USA). Antibody of cyclin A2, Apf-1 and cytochrome C, p21, p27, and p53 were purchased from Cellsignaling (USA). The total RNA isolation kit and the two-step RT-PCR kit were purchased from Tiangen. All tumor cell lines were obtained from the Shanghai Institute for Biological Science (China). Purity of complexes **1–3** and **OD** were >98%, and were dissolved in DMSO for the preparation of stock solution at a concentration of 2.0 × 10^−3^ M. Tris-NaCl buffer solution (5 mM Tris, 50 mM NaCl, adjusted to pH = 7.35 by hydrochloric acid) was prepared using double distilled water.

The PerkinElmer FT-IR Spectrometer was used to examine IR spectra; PerkinElmer Series II CHNS/O 2400 elemental analyzer was used for C, H and N elemental analyses; Bruker Daltonics HCT ESI-MS recorded mass spectra; Bruker AV-500 NMR spectrometer examined compounds’ NMR spectra; Shimadzu RF-5301/PC spectrofluorophotometer measured the fluorescence; M1000 microplate reader (Tecan Trading Co. Ltd., Shanghai, China) was used in MTT assay and fluorescent intercalator displacement (FID) assay; FACS Aria II flow cytometer (BD Biosciences, San Jose, CA, USA) was used in cell cycle analysis; The 7500 fast Real Time PCR (ABI Co. Ltd., USA) was used in FRET assay. The crystal structures have been determined by single crystal X-ray diffraction performed on a Bruker Smart Aapex2 CCD.

### Synthesis and characterization of OD and **1–3**

#### Synthesis and characterization of OD

The ligand **OD** was synthesized according to the report^66^. (+)-Boldine (0.005 mol, 1.637 g) and 2-hydrazino-2-imidazoline were used. and the mixture was dried over anhydrous sodium sulfate, condensed under reduced pressure. The crude solid was purified by column chromatography using chloroform/methanol (100:1~80:1) elution to afford the product **OD** as a red solid in a yield of about 43%. ESI-MS m/z: 366 [M + H]^+^. ^1^H NMR (500 MHz, DMSO-*d*_*6*_) δ 8.8 (d, 1H, 11-H), 8.73 (s, 1H, 5-H), 8.0 (d, 1H, 4-H), 7.73 (s, 1H, 8-H), 7.64 (s, 1H, 3-H), 4.95 (m, 1H, OCH), 4.00 (s, 3H, CH_3_), 3.99 (s, 3H, CH_3_),1.45 (d, 6H, 2 × CH_3_). Elemental analysis, calcd (%) for C_21_H_19_NO_5_: C 69.03, H 5.24, N 3.83,O 21.89; found: C 69.10, H 5.18, N 3.86, O 21.86. IR (KBr): 3425, 2980, 1651, 1594, 1418, 1386, 1351, 1280, 1214, 1150, 932 cm^−1^.

#### Synthesis of [Co(OD)_2_(H_2_O)_2_](ClO_4_)_2_ (1)

**OD** (0.03 mmol, 0.0109 g), methanol (1.5 mL) and chloroform (0.5 mL) were placed in a thick Pyrex tube (ca 20 cm long). The mixture was frozen by liquid N_2_, followed by addition of Co(ClO_4_)_2_ · 6H_2_O (0.06 mmol, 0.0223 g), evacuated under vacuum and sealed. Then it was heated at 80 °C for 72 h. After cooling, the dark red rod-like crystals suitable for X-ray single-crystal diffraction analysis were isolated in 60% yield. ESI-MS m/z: 824.14, [Co(OD)_2_ + (H_2_O) + OH]^+^; 888.113, [Co(OD)_2_ + ClO_4_]^+^, 1139.29, [Co(OD)_2_ + 4(H_2_O) + 2ClO_4_ + DMSO + H]^+^ (See Figure S12); Elemental analysis, calcd (%) for C_42_H_42_Cl_2_CoN_2_O_20_: C 49.23, H 4.13, N 2.73, O 31.23; found: C 49.21, H 4.15, N 2.71, O 31.26. IR(KBr): (−OH) 3412.25(vs), (Ar−H) 2978 (m), (C=O) 1574 (m), (C=C) 1539, 1506, 1464, 1418 (s), (ClO_4_^−^) 1108 (s), (C−O) 1287 1256, (C−N) 1015 (m) cm^−1^ (See Figure S13).

#### Synthesis of [Ni(OD)_2_(CH_3_OH)_2_](ClO_4_)_2_ (2)

**OD** (0.03 mmol, 0.0109 g), methanol (1.5 mL) and H_2_O (0.3 mL) were placed in a thick Pyrex tube (ca 20 cm long). The mixture was frozen by liquid N_2_, followed by addition of Ni(ClO_4_)_2_ · 6H_2_O (0.06 mmol, 0.0219 g), evacuated under vacuum and sealed. Then it was heated at 80 °C for 72 h. After cooling, the dark red rod-like crystals suitable for single crystal X-ray diffraction analysis were isolated in 50% yield. ESI-MS m/z: 1177.29, [Ni(OD)_2_ + 3H_2_O + 3DMSO + ClO_4_]^+^; 1080.65, [Ni(OD)_2_ + 4H_2_O + 2DMSO + 2CH_3_OH]^2+^ (See Figure S14); elemental analysis, calcd (%) for C_44_H_46_Cl_2_NiN_2_O_20_: C 50.21, H 4.41, N 2.66, O 30.40; found: C 50.26, H 4.37, N 2.68, O 30.42. IR(KBr): (−OH) 3427(vs), (Ar−H) 2979(m), (C=O) 1573 (m), (C=C) 1506, 1466, 1416, 1416 (s), (ClO_4_^−^) 1110 (s), (C−O) 1286, 1255, (C−N) 1072 (m) cm^−1^ (See Figure S15).

#### Synthesis of [Zn(OD)_2_(H_2_O)_2_](ClO_4_)_2_ (3)

**OD** (0.03 mmol, 0.0109 g), methanol (1.5 mL) and chloroform (0.5 mL) were placed in a thick Pyrex tube (ca 20 cm long), the mixture was frozen by liquid N_2_, followed by addition of Zn(ClO_4_)_2_ · 6H_2_O (0.06 mmol, 0.0219 g), evacuated under vacuum and sealed. Then it was heated at 80 °C for 72 h. After cooling, the dark red rod-like crystals suitable for X-ray single-crystal diffraction analysis were isolated in 50% yield. ESI-MS m/z: 1144.28, [Zn(OD)_2_ + 4H_2_O + 2ClO_4_ + DMSO + H]^+^; 1260.25, [Zn(OD)_2_ + 6H_2_O + 2ClO_4_ + 2DMSO + H]^+^ (See Figure S16), Elemental analysis, calcd (%) for C_42_H_42_Cl_2_ZnN_2_O_20_: C 48.92, H 4.11, N 2.72, O 31.03; found: C 48.86, H 4.16, N 2.68, O 31.08. IR(KBr): (−OH) 3428(vs), (Ar−H) 2979(m), (C=O) 1576 (m), (C=C) 1503, 1465, 1421 (s), (ClO_4_^−^) 1107 (s), (C−O) 1288 1256, (C−N) 1107(m) cm^−1^ (See Figure S17).

### X-ray Crystallography

The data collection of single crystal of **OD** and **1–3** was performed on an Bruker Smart Aapex2 CCD and Rigaku Saturn CCD diffractometer equipped with graphite monochromated Mo-Kα radiation (λ = 0.7107 Å) at room temperature. The structure was solved with direct methods and refined using OLEX2 and SHELXL-97 programs^67^. The non-hydrogen atoms were located in successive difference Fourier synthesis. The final refinement was performed by full-matrix least-squares methods with anisotropic thermal parameters for no-hydrogen atoms on *F*^*2*^. The hydrogen atoms were added theoretically and riding on the concerned atoms. The parameters used intensity collection and refinements are summarized in Tables S1, The selected bond lengths and angles of **1–3** are given in Table S2.

### Solution Stability

The stability of **1–3** under physiological conditions (Tris-KCl buffer solution with pH value of 7.35, containing 1% DMSO), was carried out using UV-vis spectroscopy and HPLC. **1–3** were dissolved in DMSO at 1 × 10^−3^ M, and then diluted by buffer solution. The UV-vis absorbance of these complexes at 0 h, 12 h and 48 h were measured, respectively. The solution stability of **3** was further confirmed by HPLC experiments. **3** in aqueous solution at 1.0 mg · mL^−1^ was analyzed by HPLC at 0 h and 24 h respectively, with reversed-phase C18 column and methol/H_2_O (80:20) as mobile phase.

### Cell Lines and Culture

The human tumor cell lines (BEL-7404, HepG2, MGC80–3, T-24, SK-OV-3/DDP and HeLa cells) and the human normal liver cell line HL-7702 were obtained from Shanghai Cell Bank in Chinese Academy of Sciences. Cells were cultured in Dulbecco’s modified Eagle’s medium (DMEM) or RPMI-1640 medium, which supplemented with 10% fetal bovine serum (FBS) and 100 U/mL penicillin and streptomycin. Cells were cultured at 37 °C in a humidified atmosphere with 5% CO_2_/95% air. Tested compounds (2.0 mM) were prepared as DMSO stock solution before they were diluted to working concentrations by PBS buffer (containing 0.5% DMSO), while cisplatin dissolved in 0.9% sodium chloride solution was used as positive controls.

### MTT Assay

Cells in their logarithmic phase were seeded in 96-well plates with 5.5 × 10^3^/180 μL per well for 24 h. Then 20 μL of compounds at various concentrations were added to each well to achieve final concentrations of 1.25, 2.5, 5.0, 10.0, and 20.0 μM. All the cells were incubated with test compounds for another 48 h before MTT assay. Control wells contained media with 0.5% DMSO. After 48 h of cultivation, 10 μL of MTT (5 mg/mL in PBS) was added to each well, and cells were incubated at 37 °C for 6 h. The contents in the wells were pipetted out carefully, and the formazan crystals formed were dissolved in 100 μL of DMSO, and the absorbance was measured by enzyme labeling instrument with 490 nm/630 nm double wavelength measurement. The IC_50_ value was defined as a drug concentration killing 50% cells in comparison with untreated controls, which was calculated by the Bliss method (n = 5). All tests were repeated in at least three independent trials.

### Flow Cytometric Analysis

Cell cycle progression and apoptosis were determined by flow cytometric analysis. (a) Cell cycle arrest assay: HepG2 cells in their logarithmic phase were plated into 50 mL plates at a final density of 5−6 × 10^6^ cells/well overnight, then they were treated with various doses (0.25, 0.5 and 1.0 μM) of **1–3** for another 24 h. Cells were trypsinized, collected, and fixed in ice-cold 75% ethanol at −20 °C overnight. After centrifugation, the fixed cells were rinsed with ice-cold phosphate buffered saline (PBS), and resuspended in 0.5 mL of PBS containing 100 μg/mL RNase, 50 μg/mL propidium iodide (PI) in the dark for 5−10 min. The cell cycle distribution was analyzed by FACS Calibur flow cytometer (BD) and calculated using ModFIT LT software. (b) Apoptosis assay: 1 × 10^5^ HepG2 cells were plated in 6-well plates, after incubation for 24 h with complexes **1–3** at 1.0, 2.0 and 5.0 μM, cells were collected, and Apoptosis Detection kit FITC Annexin V was used to analyze the cell apoptosis according to the manufacturer’s instructions.

### Fluorescence Morphological Examination

To examine **1–3** and **OD** inducing HepG2 cells apoptosis, Hoechst 33258 staining and AO/EB staining were used to detect the cellular apoptotic morphological changes. 1 × 10^6^ HepG2 cells were seeded in six-well plates, and treated with 0, 1.0 μM of **OD** or **1–3** for 24 h, respectively. For Hoechst 33258 staining, the cells after treatment were fixed on a plate for 10 min at room temperature and then stained with Hoechst 33258 for another 10 min in the dark after removal of the fixed liquid. After washing with PBS three times, the HepG2 morphological features of apoptosis were captured by fluorescence microscope with excitation wavelength of 330−380 nm. Apoptotic cells were defined based on the changes of nuclear morphology, such as chromatin condensation and fragmentation. For AO/EB staining assay, after incubation with compounds, the cells were trypsinized and harvested, suspended in PBS, stained by 100 μg/mL of AO/EB for 10 min at room temperature, and then visualized by the fluorescence microscopy at the excitation wavelength of 545 nm. About 200 cells in random were assayed.

### Comet Assay for DNA Damage

The comet assay is a simple method for measuring DNA strand breaks in cells. The comet assay reagent kit was purchased from Trevigen, and the experiment was carried out according to the manufacturer’s instructions. After treatment with **OD** and **1–3** at 1.0 μM, HepG2 cells were harvested and suspended at 1 × 10^6^ Hep-G2 cells/mL in PBS. The cells suspension was mixed with melted LM agarose at a ratio of 1:10 (v/v). An aliquot (75 μL) of the mixture was immediately pipetted onto the slide (Comet Slide TM). After refrigeration for 30 min, the slide was immersed in pre-chilled lysis solution and left on ice for 60 min, followed by immersing in freshly prepared alkaline solution (300 mM NaOH, 1 mM EDTA, pH > 13) for 60 min on ice in darkness. After DNA unwinding, the slide was subjected to alkaline solution for electrophoresis in a Savant ps 250 system set at 300 mA and 1 volt/cm for 30 min. After electrophoresis, the slide was rinsed with distilled H_2_O, fixed in 70% ethanol for 5 min and air-dried overnight. DNA was stained with SYBR Green I (Trevigen) and visualized under a fluorescence microscope (Nikon, Eclipse E600).

### Mitochondrial Membrane Potential (MMP) Measurements (ΔΨm)

The changes in the MMP were evaluated by using a JC-1 assay, 1 × 10^6^ HepG2 cells were seeded in a 6-well culture plate and incubated at 37 °C for 24 h, then the cells were exposed to **OD** and **1–3** at 1.0 μM in media and incubated for another 24 h. After the treatment, the medium was removed, and then the JC-1 staining (5 μg/mL) was added and incubated for another 20 min at 37 °C. The medium was removed again and cells were washed twice with cold PBS in dark, finally HepG2 cells were suspended in PBS and examined under the fluorescence microscopy. The emission fluorescence for JC-1 was monitored at 530 and 590 nm, under the excitation wavelength at 488 nm.

### Assay of Reactive Oxygen Species (ROS) Generation

The DCFH-DA method was used to determined the effects of **1–3** on ROS generation as reported^68^. In this method, the stable non-fluorescent DCFH-DA was hydrolyzed by esterases in cells to non-fluorescent DCFH, which was rapidly oxidized by ROS to highly fluorescent 2′,7′-dichlorofluorescein (DCF). Therefore, the fluorescence intensity of DCF is proportional to the amount of ROS produced in the cells. In this experiment, 1 × 10^6^ HepG2 cells were treated with 1.0 μM of **1–3** and **OD** for 24 h, and then cells were harvested, washed with ice-cold PBS once and incubated with 100 μM of DCFH-DA (100 μM in a final concentration) at 37 °C for 15 min in the dark. After being incubated with DCFH-DA, the cells were washed again and 1 mL PBS was added. The image of ROS generation was visualized by using a fluorescence microscope with the excitation wavelength at 488 nm. The ROS generation was assessed using flow cytometry by examining the fluorescence of DCF in cells for each sample collected from 1 × 10^6^ cells.

### Measurement of Intracellular Ca^2+^ Levels

The intracellular Ca^2+^ levels in **1–3**-treated HepG2 cells were determined by using a fluorescent dye Fluo-3 AM, which could cross the cell membrane and be cut into Fluo-3 by intracellular esterase. The Fluo-3 can specifically bind to Ca^2+^ and has a strong fluorescence with an excitation wavelength of 488 nm. After exposed to **1–3** at a concentration of 1.0 μM for 24 h, the HepG2 cells were harvested and washed with PBS twice, then re-suspended in Fluo-3 AM (5.0 μM) for 30 min in dark. Detection of intracellular Ca^2+^ was carried by Flow cytometer at 525 nm excitation wavelength.

### Examine Release of Cytochrome C by Immunofluorescence

HepG2 cells were grown on polylysine-coated coverslips. HepG2 cells were treated with staining solution for 60 min after treatment with **OD** and **1–3**, washed with PBS three time, and then blocked in antifade mounting medium for 30 min at room temperature. The coverslips were incubated with primary antibody buffer (1:1000) for 3 h at 25 °C. The cover slips were washed and incubated with Cy3 secondary antibodies (1:2000 for 1 h at 25 °C. Finally, the cells were stained with 0.1 mg/mL DAPI. Fluorescence images were captured using confocal microscopy (CarlZeiss LSM 710, Germany).

### Caspase-3, -8 and -9 Activity Determinations by Flow Cytometry

The measurement of caspase-3, -8 and -9’s activities was performed by CaspGLOW fluorescein active caspase-3, -8 and -9 staining kit. HepG2 cells were treated with 1 μM **1–3** for 24 h, harvested at a density of 1 × 10^6^ cells/mL, and then suspended in 300 μL volume with PBS. To the suspension, 1 μL of caspase-3 inhibitor (FITC-DEVD-FMK), caspase-8 inhibitor (FITC-IETD-FMK) or caspase-9 inhibitor (FITC-LEHD-FMK) was consequently added and incubated for 1 h at 37 °C in 5% CO_2_ incubator. The cells were then examined by a FACSAria II flow cytometer equipped with a 488 nm argon laser. The results were represented as the percent change on the activity compared with the control.

### Western Blotting Analysis

After incubated with **1–3** (1.0 μM) for 24 h, HepG2 cells were harvested and lysed in 150 μL of lysed buffer (149 μL RIPA and 1 μL PMSF), and then placed on ice for 15 min. The total proteins were extracted by centrifuging the cell sample at 10000 rpm at 4 °C for 10 min. Then about 50 μg of purified proteins were mixed with equal volume of electrophoresis sample buffer, and the mixture was boiled for 10 min. Meanwhile the equal protein content from the control cells was prepared in the same way. The cell sample (10 μL) was loaded onto 10% SDS-PAGE gels and then transferred onto a microporous polyvinylidene difluoride (PVDF) membrane. The membrane was blocked with 5% BSA in TBST buffer for more than 2 h. After the removal of TBST buffer, membranes were incubated with an appropriate dilution of specific primary antibodies in TBST overnight at 4 °C and then washed with TBST three times. The membranes were incubate with secondary antibodies for 1 h, and washed with TBST. The immunoreactive signals were detected using an enhanced chemo-luminance kit (Pierce ECL Western Blotting Substrate) following the procedures given in the user manual.

### RNA Extraction and Real-Time Quantitative PCR

HepG2 cells were seeded in flasks and exposed to **1–3** at 1.0 μM for 24 h. These cells were harvested from each well and were lysed using TRIZol reagent, and total mRNA were extracted from cells using RNA kit (TIANGEN) according to manufacturer’s protocol. Reverse transcription was carried out using cDNA reverse transcription kits (BioRT Two Step RT-PCR Kit), and the synthesized cDNA was stored at −80 °C. Real-time PCR was performed on 7500 fast Real Time PCR (ABI Co. Ltd., USA) by using 2.5 × Real Master Mix/20 × SYBR solution (TIANGEN). The primer sequences were shown in Table S4. The total volume of 20 μL of RT-PCR reaction mixtures contained 10.0 μL of 2 × Mltra SYBR Mixture (With ROX) solution, 0.5 μM of each forward and reverse primers, 1.0 μL of template DNA, and 8.0 μL nuclease-free water. The program used for all genes was consisted of a denaturing cycle of 10 min at 95 °C, 40 cycles of PCR (95 °C for 15 s, 58 °C for 30 s, and 72 °C for 30 s), The specificity of the RT-PCR product was confirmed by melting curve analysis. The sizes of PCR products were confirmed by agarose gel electrophoresis and ethidium bromide staining. Three replications were performed, and the values obtained for the target gene expression were normalized to GAPDH. The values obtained for tested gene expression were analyzed by the relative gene expression 2^−ΔΔCT^ method in the program Origin 8.0, where −ΔΔCT = (CT_target_ − CT_GAPDH_)_unknown_ − (CT_target_ − CT_GAPDH_)_calibrator_^69^.

### *In Vivo* Tumor Growth Inhibition Experiment

BALB/c nude mice (male, 17–20 g, 5–6 weeks old) were supplied by Guangxi Medical University Laboratory Animal Centre (Guangxi, China, approval no. SCXK 2014–0002 and SYXK 2014–0003). All of the experimental procedures were carried out according to the NIH Guidelines for the Care and Use of Laboratory Animals. Animal experiments were approved by the Animal Care and Use Committee of Guangxi Medical University. Animal were housed at IVC system (individual ventilated caging) with conditions of constant photoperiod (12 h light/12 h dark at 25−28 °C and 45−65% humidity). The xenograft tumor model was established in the same way as we reported before^30^. The maximum solubility of **3** in DMSO/saline solvent (v/v, 5/100) with administration volume 0.8 mL/20 g was used as the high dose (6.0 mg/kg) group (n = 6). Meanwhile low dose group mice got 3.0 mg/kg, intraperitoneal injection every day, and the control group received corresponding solvent. The positive control group was administered with cisplatin at 2.0 mg/kg every two days by intraperitoneal injection (cisplatin dissolved in saline).

The tumor volumes and body weight were measured every 3 days, and tumor size was calculated as volume = (w^2^*l*)/2, where *w* is the width and *l* is the length in mm of tumor^70^. On day 21, the mice were sacrificed, and the tumor tissue was dissected out and weighted. The *in vivo* antitumor activity is evaluated by two indicators^71^,^72^: the tumor relative increment rate (T/C = T_RTV_/C_RTV_ × 100%) and tumor weight inhibition rate [IR = (W_c_ − W_t_)/W_c_ × 100%,], where T_RTV_ and C_RTV_ were the RTV of treated group and control group, respectively (RTV: relative tumor volume, RTV = V_t_ /V_0_); W_t_ and W_c_ are the average tumor weight of complex-treated and vehicle controlled group, respectively.

## Additional Information

**How to cite this article:** Qin, J.-L. *et al*. Oxoaporphine Metal Complexes (Co^II^, Ni^II^, Zn^II^) with High Anti-tumor Activity by Inducing Mitochondria-Mediated Apoptosis and S-phase Arrest in HepG2. *Sci. Rep.* **7**, 46056; doi: 10.1038/srep46056 (2017).

**Publisher's note:** Springer Nature remains neutral with regard to jurisdictional claims in published maps and institutional affiliations.

## Supplementary Material

Supplementary Information

## Figures and Tables

**Table 1:** The antitumor activity of oxoaporphine and oxoisoaporphine’s Co(II), Ni(II) and Zn(II) complexes.

Complexes	Structures	Antitumor activity: IC_50_ (μM)	Targets
Co(II) complexes	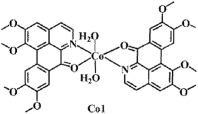	MCF-7: 5.1 ± 0.8 HeLa: 11.4 ± 5.0 BEL-7404:13.1 ± 8.1	Bind to DNA via intercalation; TOPO I inhibitor; arrest cell-cycle at the S phase^27^.
	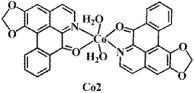	A2780: 2.2 ± 0.5 HT-29: 1.7 ± 0.3 HeLa: 6.4 ± 0.7	Bind to DNA via intercalation; TOPO I inhibitor (≤10 μM)^28^.
	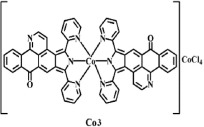	SK-OV-3/DDP: 1.0 ± 0.8 T-24: 3.1 ± 0.8 Hep-G2: 4.3 ± 1.1 BEL-7402: 9.1 ± 0.9 NCI-H460: 9.4 ± 0.6	telomerase inhibitor, targeting G-quadruplex; caused S phase arrest; inhibition BEL-7402 tumor growth with 62.1% *in vivo*^29^.
Ni(II) complexes	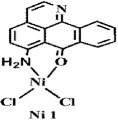	Hep-G2: 8.24 ± 0.76 SK-OV-3: 10.21 ± 1.23 NCI-H460: 10.79 ± 2.01	**Ni1** and **Ni2** were telomerase inhibitors, targeting c-myc, telomeric, bcl-2 and G-quadruplexes; caused telomere/DNA damage and S phase arrest^30^.
	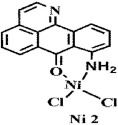	Hep-G2: 18.16 ± 0.79 SK-OV-3: 15.04 ± 0.92 NCI-H460: 15.64 ± 0.83	
	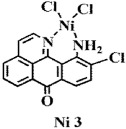	Hep-G2: 28.76 ± 1.42 SK-OV-3: 29.06 ± 0.82 NCI-H460: 35.18 ± 1.49	As the low antitumor activity of **Ni3**, further mechanism study were not carried out^30^.
Zn(II) complexes	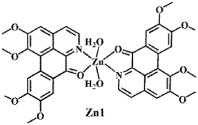	HeLa: 16.4 ± 4.5	Bind to DNA via intercalation; TOPO I inhibitor; arrest cell-cycle at the S phase^27^.
	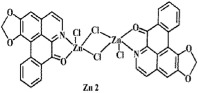	HT-29: 5.8 ± 1.2 A2780: 6.7 ± 0.8 BEL-7404: 8.4 ± 0.4	Intercalatly and covalently bind to DNA; TOPO I inhibitor^28^.
	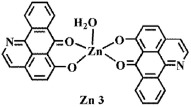	T-24: 8.19 ± 1.81 MGC80-3: 10.29 ± 0.67 BEL-7404: 10.91 ± 0.14	up-regulated p27, p21, p53 and CDK2 proteins; down-regulated chk1, chk2, cdc25A and cyclin E proteins^31^.

**Table 2:** IC_50_ (μM) values of **OD**, **1**–**3** for the selected cells for 48 h.

Compounds	HepG2	T-24	BEL-7404	MGC80-3	SK-OV-3/DDP	HL-7702
**OD**	8.98 ± 0.76	9.12 ± 0.11	24.77 ± 1.02	23.28 ± 0.88	38.53 ± 1.42	9.28 ± 0.43
**1**	0.66 ± 0.11	0.45 ± 0.17	8.98 ± 0.15	1.38 ± 0.14	0.26 ± 0.03	8.11 ± 0.55
**2**	0.20 ± 0.09	0.23 ± 0.13	9.66 ± 0.35	2.86 ± 0.29	0.18 ± 0.03	5.35 ± 0.34
**3**	0.39 ± 0.03	0.51 ± 0.17	12.64 ± 0.45	4.31 ± 0.31	0.57 ± 0.07	7.13 ± 0.46
cisplatin	7.72 ± 0.83	7.48 ± 0.26	10.01 ± 0.53	15.97 ± 1.53	65.97 ± 1.53	5.18 ± 0.68

**Table 3:** DNA damage induced by **1**–**3** (1.0 μM) in HepG2 cells detected by comet assay.

	Comet length	Tail length	Tail moment	Olive tail moment
OD	49.45 ± 6.19	1.96 ± 1.78	0.12 ± 0.21	0.13 ± 0.29
1	65.45 ± 6.54*	8.01 ± 4.98**	4.65 ± 1.34**	3.67 ± 2.09**
2	99.56 ± 10.57**	45.69 ± 8.27**	29.52 ± 8.66**	20.56 ± 7.54**
3	119.12 ± 6.59*	58.67 ± 9.14**	30.46 ± 6.98	22.67 ± 3.76**

Compared with the control, *P < 0.05; **P < 0.01.
