# Supplementary material for: Oxoaporphine Metal Complexes (CoII, NiII, ZnII) with High Antitumor Activity by Inducing Mitochondria-Mediated Apoptosis and S-phase Arrest in HepG2
Source: Sci Rep. 2017 Apr 24;7:46056. doi: 10.1038/srep46056 (PMC5402304; doi:10.1038/srep46056)
Supplement: Supplementary Information [file srep46056-s1.pdf]

OxoaporphineMetal Complexes (Co^{II}, Ni^{II}, Zn^{II}) with High Antitumor Activity by InducingMitochondria-Mediated Apoptosis and S-phase Arrest in HepG2

Jiao-Lan Qin[†] *, Wen-Ying Shen*[†] *, Zhen-Feng Chen*, Li-Fang Zhao, Qi-Pin Qin, Yan-Cheng Yu, Hong Liang**

State Key Laboratory for Chemistry and Molecular Engineering of Medicinal Resources, School of Chemistry and Pharmacy, Guangxi Normal University, 15 Yucai Road, Guilin 541004, P. R. China.

Table S1 Crystallographic data and refinements of complexes **1−3** and **OD.**

	**OD**	**1**	**2**	**3**
Formula	$C_{21}H_{19}NO_5$	$C_{42}H_{42}Cl_2CoN_2O_{21}$	$C_{44}H_{46}C_{l2}NiN_2O_{20}$	$C_{42}H_{46}Cl_2ZnN_2O_{22}$
M_r	365.38	1040.61	1052.44	1067.08
Crystal system	*Monoclinic*	*Triclinic*	*monoclinic*	*Triclinic*
Space group	*P2(1)/c*	*P-1*	*P2(1)/n*	*P–1*
a/Å	14.7970(13)	11.9385(3)	7.9782(7)	11.9525(7)
b/Å	8.9351(8)	12.6488(3)	15.9908(17)	12.6917(4)
c/Å	15.8677(16)	15.9365(4)	35.913(5)	16.0413(9)
α/°	90.00(3)	74.630(2)	90.00	74.647(4)
β/°	107.0190(10)	79.728(2)	89.742(10)	79.932(5)
γ/°	90.00(2)	87.418(2)	90.00	87.379(4)
V/Å^3	2006.0(3)	2283.28 (10)	4581.6(9)	2310.5(2)
T/K	293(2)	293(2)	293(2)	293(2)
Z	4	2	4	2
D_c/g cm^{-3}	1.263	1.514	1.526	1.534
θ/°	2.65 to 25.02	2.82 to 26.37	2.83 to 28.63	2.93 to 26.37
F(000)	804	1074	2184	1104
μ(Mo Kα)/mm^{-1}	0.091	0.578	0.624	0.734
Total no. reflns	10136	9319	4140	9408
No. indep. reflns	3525	8004	2839	6074
R1 [$I > 2\sigma(I)$]	0.0766	0.0585	0.0758	0.0686
$wR2$(all data)	0.2175	0.1736	0.2037	0.1084
Gof(F^2)	1.084	1.085	1.078	1.034

Table S2 Selected bond lengths[Å] and angles [°]of **OD** and **1−3**

OD					
N(1)–C(1)	1.342(4)	O(4)–C(14)	1.370(4)	O(1)–C(2)–C(3)	121.9(3)
N(1)–C(13)	1.345(5)	O(5)–C(15)	1.352(4)	O(1)–C(2)–C(1)	120.5(3)
O(1)–C(2)	1.240(3)	O(5)–C(19)	1.444(4)	O(4)–C(14)–C(9)	120.2(3)
O(2)–C(5)	1.355(4)	O(6)–C(22)	1.43(8)	O(2)–C(5)–C(6)	116.9(3)
O(2)–H(2)	0.8200	C(1)–N(1)–C(13)	118.1(3)	O(3)–C(6)–C(7)	124.8(3)
O(3)–C(6)	1.363(4)	N(1)–C(1)–C(10)	123.6(3)	N(1)–C(13)–C(12)	123.7(4)
O(3)–C(17)	1.429(4)	C(6)–O(3)–C(17)	117.3(3)	N(1)–C(1)–C(2)	116.3(3)
O(4)–C(18)	1.310(5)	C(18)–O(4)–C(14)	127.4(4)	O(2)–C(5)–C(4)	124.6(3)
**Complex 1**					
Co(1)–O(1)	2.138(2)	O(6)–Co(1)–O(1)	175.90(9)	C(6)–O(1)–Co(1)	114.09(18)
Co(1)–O(6)	2.091(2)	O(6)–Co(1)–O(11)	87.73(11)	C(30)–O(6)–Co(1)	114.33(18)
Co(1)–O(11)	2.110(3)	O(6)–Co(1)–N(2)	78.17(9)	C(1)–N(1)–Co(1)	126.7(2)

Co(1)–O(12)	2.052(3)	O(11)–Co(1)–O(1)	88.61(10)	C(5)–N(1)–Co(1)	114.82(18)
Co(1)–N(1)	2.081(2)	O(12)–Co(1)–O(1)	92.27(12)	N(2)–Co(1)–O(11)	89.98(11)
Co(1)–N(2)	2.100(3)	O(12)–Co(1)–O(6)	91.36(12)	C(22)–N(2)–Co(1)	113.06(19)
N(1)–C(1)	1.349(4)	O(12)–Co(1)–O(11)	178.80(13)	N(1)–C(1)–C(2)	121.9(3)
N(1)–C(5)	1.341(4)	O(12)–Co(1)–N(1)	92.50(11)	C(23)–N(2)–Co(1)	129.2(2)
N(2)–C(22)	1.337(4)	O(12)–Co(1)–N(2)	90.61(11)	N(1)–Co(1)–O(11)	86.89(10)
N(2)–C(23)	1.338(4)	N(1)–Co(1)–O(1)	77.33(8)	N(1)–Co(1)–N(2)	176.70(10)
O(6)–C(30)	1.248(3)	N(1)–Co(1)–O(6)	100.62(9)	N(2)–Co(1)–O(1)	103.68(9)
**Complex 2**					
Ni(1)–O(1)	2.047(3)	O(1)–Ni(1)–O(1A)	180.00(11)	C(1)–N(1)–Ni(1)	129.8(4)
Ni(1)–O(10)	2.059(4)	O(1)–Ni(1)–O(10)	89.50(18)	C(14)–N(1)–Ni(1)	112.2(3)
O(1)–C(15)	1.253(6)	O(1)–Ni(1)–O(10A)	90.50(18)	C(15)–O(1)–Ni(1)	113.0(3)
N(1)–C(1)	1.353(6)	O(10)–Ni(1)–O(10A)	180.0(3)	N(1)–C(1)–C(2)	122.3(5)
N(1)–C(14)	1.341(6)	N(1)–Ni(1)–O(1A)	99.18(15)	N(1)–Ni(1)–O(10A)	89.51(17)
C(1)–C(2)	1.362(7)	N(1)–Ni(1)–O(1)	80.82(15)	N(1)–Ni(1)–O(10)	90.49(17)
O(10)–C(23)	1.391(8)	N(1A)–Ni(1)–N(1A)	180.00(18)	C(23)–O(10)–Ni(1)	127.2(5)
**Complex 3**					
Zn(1)–O(1)	2.168(3)	O(6)–Zn(1)–O(1)	175.06(13)	N(2)–Zn(1)–O(12)	88.58(14)
Zn(1)–O6	2.116(3)	O(6)–Zn(1)–O(12)	87.89(14)	C(1)–O(1)–Zn(1)	112.9(2)
Zn(1)–O(11)	2.100(4)	O(11)–Zn(1)–O(1)	93.49(15)	C(2)–O(1)–Zn(1)	114.9(2)
Zn(1)–O(12)	2.160(3)	O(11)–Zn(1)–O(6)	91.19(16)	C(3)–O(1)–Zn(1)	126.3(3)
Zn(1)–N(1)	2.069(3)	O(12)–Zn(1)–O(1)	87.42(14)	C(3)–N(1)–C(2)	118.8(3)
Zn(1)–N(2)	2.080(3)	N(1)–Zn(1)–O(1)	78.00(11)	C(39)–N(2)–Zn(1)	128.6(3)
O(1)–C(1)	1.252(5)	N(1)–Zn(1)–O(6)	100.24(12)	C(40)–N(2)–Zn(1)	113.2(3)
N(1)–C(2)	1.342(5)	N(1)–Zn(1)–O(11)	92.45(14)	N(2)–Zn(1)–O(1)	102.66(12)
N(1)–C(3)	1.337(5)	N(1)–Zn(1)–O(12)	87.27(13)	N(2)–Zn(1)–O(6)	78.76(12)
N(2)–C(39)	1.352(5)	N(1)–Zn(1)–N(2)	175.77(14)	N(2)–Zn(1)–O(11)	91.69(15)

Table S3. The inhibitive ratios of OD,**1**–**3** and corresponding metal salt for the selected cells and one normal liver cell line HL-7702 for 48 h.

	HepG2	T-24	BEL-7404	MGC80-3	SKOV-3/DDP	HL-7702
OD	33.35±0.32	46.91±0.17	36.28±0.18	42.28±0.24	45.57±1.94	46.55±0.67
**1**	88.34± 1.03	85.35±0.73	63.96±0.78	61.54±0.33	78.17±1.69	56.73±0.14
**2**	87.68 ±0.92	82.31±0.60	69.38±0.65	71.86±0.28	84.44±0.65	72.34±0.22
**3**	89.58±1.22	84.16±0.76	61.00±0.19	67.30±0.21	70.80±1.69	59.26±0.15
$Co(ClO_4)_2$	32.87±0.13	36.23±0.09	23.29±0.09	24.74±0.03	21.74±0.03	45.91±0187
$Ni(ClO_4)_2$	32.57±0.29	37.86±0.19	32.31±0.07	16.35±0.09	11.25±0.12	50.14±0.06
$Zn(ClO_4)_2$	30.94±0.39	28.19±0.13	24.07±0.15.	17.63±0.07	12.33±0.05	43.18±0.19
cisplatin[b]	71.93±0.83	75.26±1.00	76.07±2.68	65.58±0.09	15.13±0.07	68.96±1.34

inhibitive ratios are represent mean ± SD of at least five independent experiments(SD represents the standard deviation). The concentration of **OD**, **1**–**3** and corresponding salts are 20μmol/L[b] cisplatin was dissolved at a concentration of 1mM in 0.154 M NaCl.

Table S4. Primer sequences used in this work.

target protein	Upstream primer sequences	Downstream primer sequences
β-Actin	AAAAGCCACCCCACTTCTCT	GACCAAAAGCCTTCATACATCTCA
caspase-8	CCAGTGCCAGACACACAGTC	CCAGCAGGTTCATGTCATC
cytochrome c	GTTCGTTGTGCCAGCGACTA	GCTTGCCTCCCTTTTCAACG
Apaf-1	AGAGGTAGCGAGTGGACGTG	CGCTGCGGCACCTCAAGTCT
p21	AGGGGACAGCAGAGGAAG	CGTTTTCGACCCTGAGAG
CDK2	AGTTACTTCTATGCCTGATTACA	TCCGTCCATCTTCATCCA
PCNA	TGAAGCACCAAACCAGGA	GCATCTCCAATATGGCTG

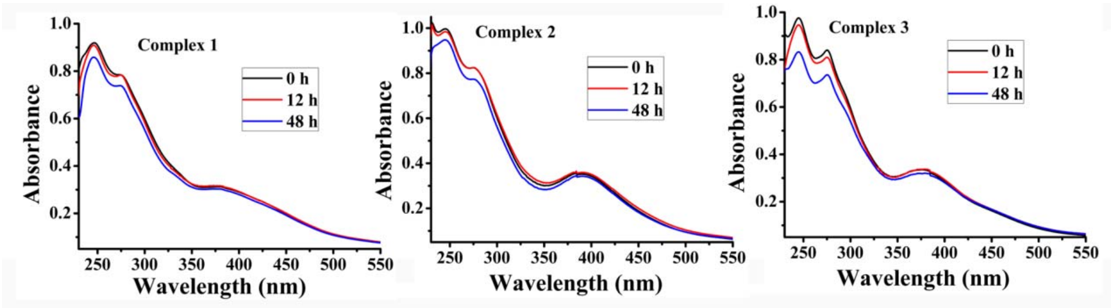

Figure S1. Solution stability of complexes **1–3** in tris buffer solution examined by UV-vis spectra.

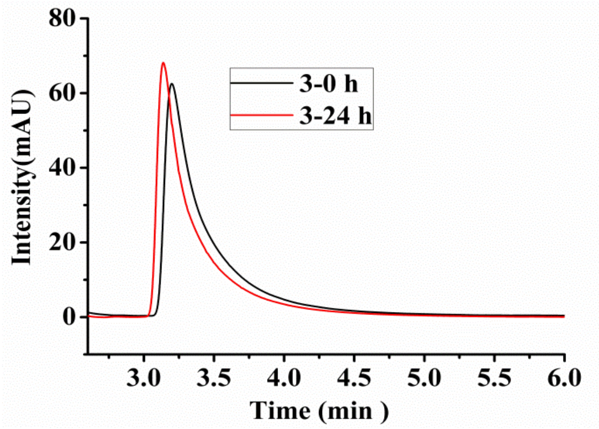

Figure S2. HPLC spectra of complex **3** in aqueous solution (1 mg/mL) with time 0 h and 24 h, respectively. Column: reversed-phase C18 column (YMC HPLC COLUMN, 250×4.6mm I. D.). Column temperature: 35ºC. Mobile phase: Methanol/H_2O (80:20). Flow rate: 1.0 ml/min. Injection volume: 10 μL.

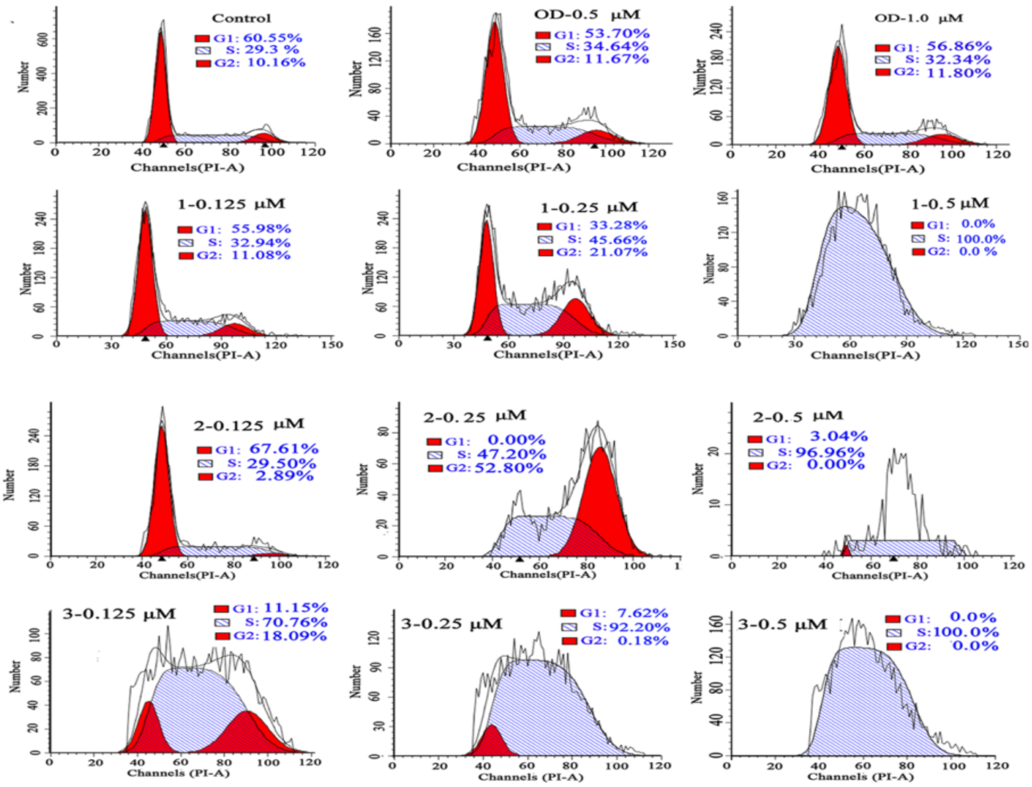

Figure S3. Cell cycle analysis of **1–3** (0.125, 0.25 and 0.5 μM) and **OD** (0.5 and 1.0 μM) for 24 h in HepG2 cells, graph bar show the distributions of cells in the different phases of cell cycle.

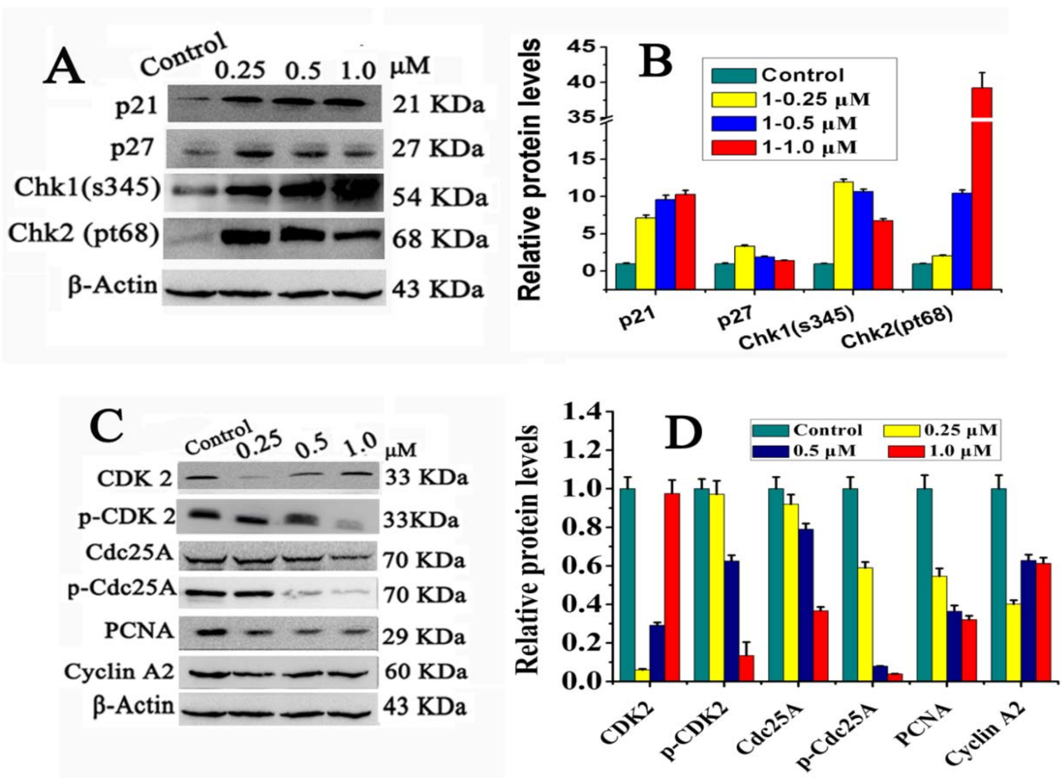

Figure S4. (A,C) Effects of complex **1** treatment in HepG2 cells on cell cycle regulatory proteins at 0.25 μM, 0.5 μM and 1.0 μM for 24 h, respectively. (B,D) The relative protein expression of each band = (density of each band/density of actin band). Mean ± SD was from three independent measurements.

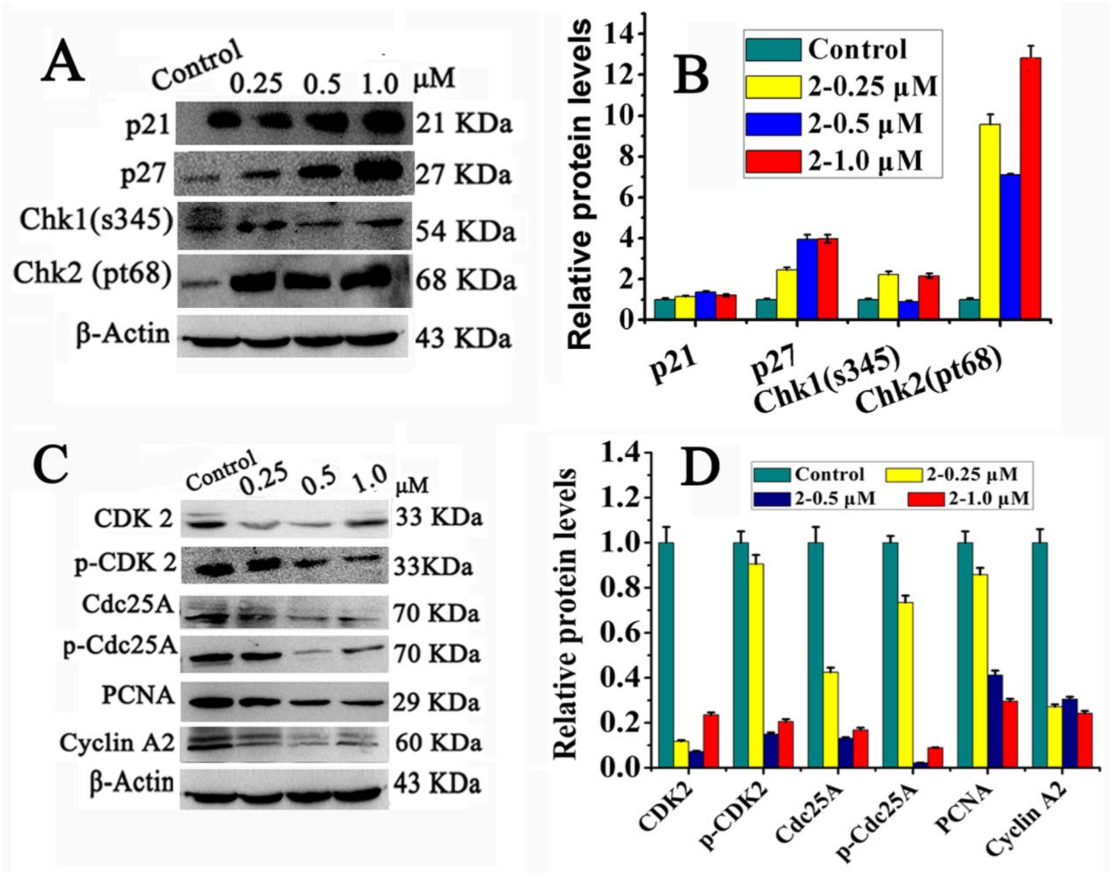

Figure S5. (a) Effects of complex **2** treatment in HepG2 cells on cell cycle regulatory proteins at 0.25 μM, 0.5 μM and 1.0 μM for 24 h, respectively.(b) The relative protein expression of each band = (density of each band/density of actin band). Mean ± SD was from three independent measurements.

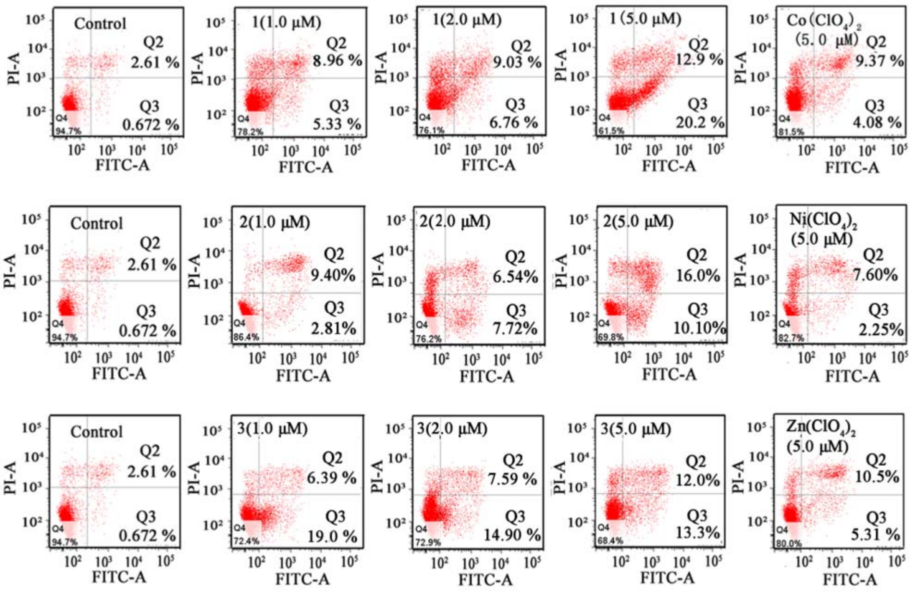

Figure S6. Effect of cell apoptosis of HepG2 treated with **1–3**, **OD** and corresponding metal salts for 24 h compared with the control cells.

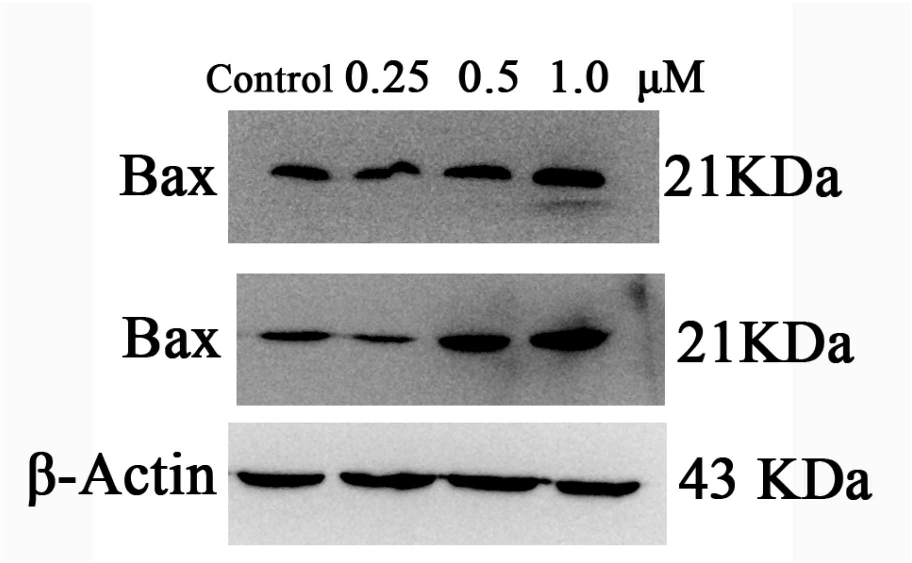

Figure S7 Multiple Western blot analysis of Bax protein after treatment of HepG2 cells with **3** at 0.25, 0.5, 1.0 μM for 24 h, respectively.

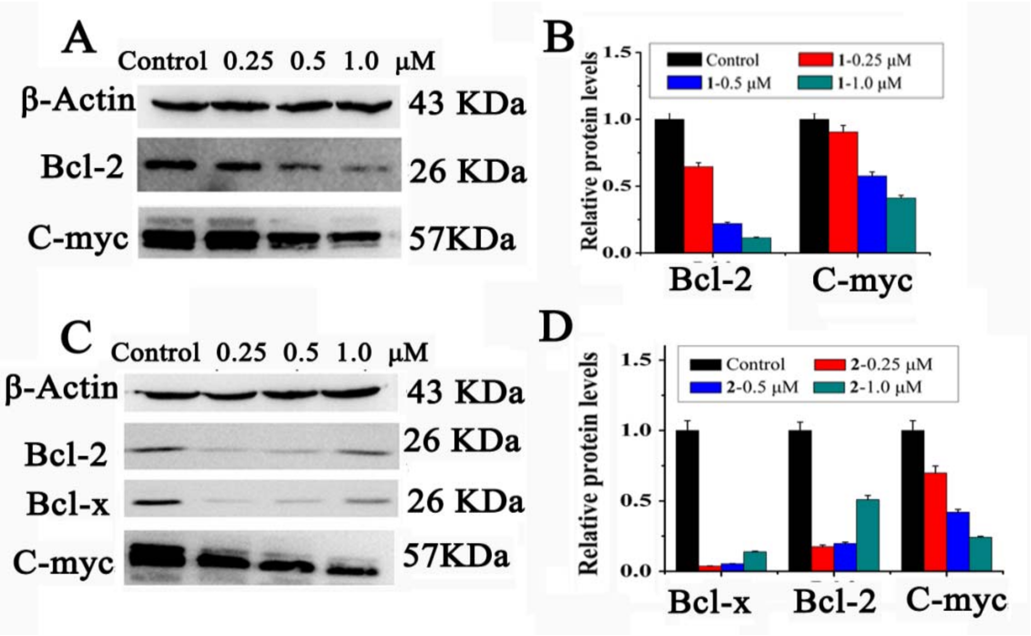

FigureS8 (a) Western blot analysis of Bcl-2, and C-myc after treatment of HepG2 cells with complex **1** at 0.25, 0.5, 1.0 μM for 24 h, respectively. (b) Densitometric analysis of Bcl-2 and C-myc band from part A. (c) Western blot analysis of Bcl-2, Bcl-xl, and C-myc after treatment of HepG2 cells with complex **2** at 0.25, 0.5, 1.0 μM for 24 h, respectively. (d) Densitometric analysis of Bcl-2, Bcl-xl and C-myc band from part C. The relative expression of each band = (density of each band/ density of actin band). Mean and SD values were from three independent measurements.

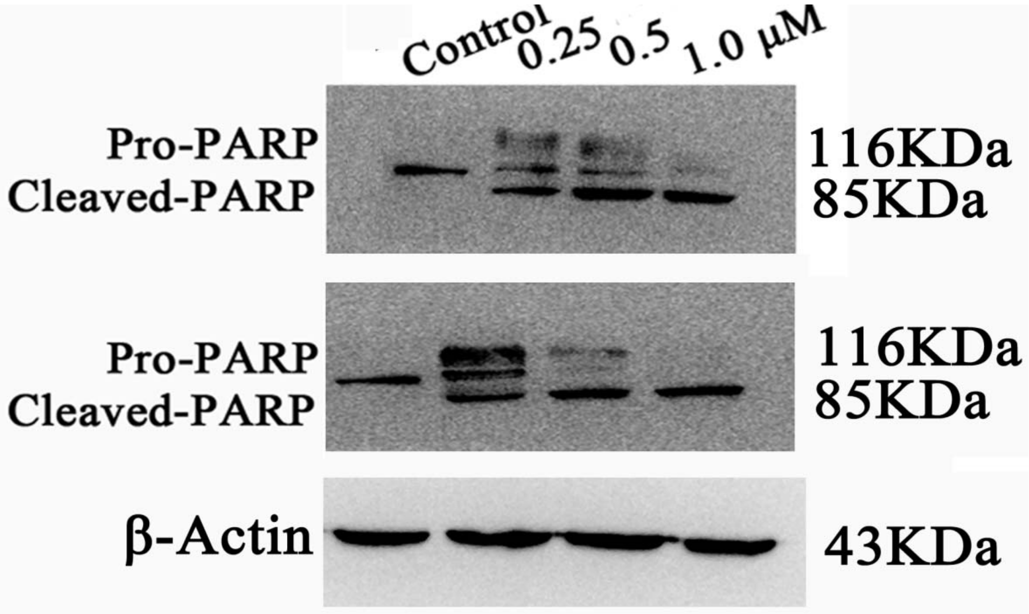

Figure S9. Multiple Western blot analysis of Pro-PARP and Cleaved-PARP proteins after treatment of HepG2 cells with **3** at 0.25, 0.5, 1.0 μM for 24 h, respectively.

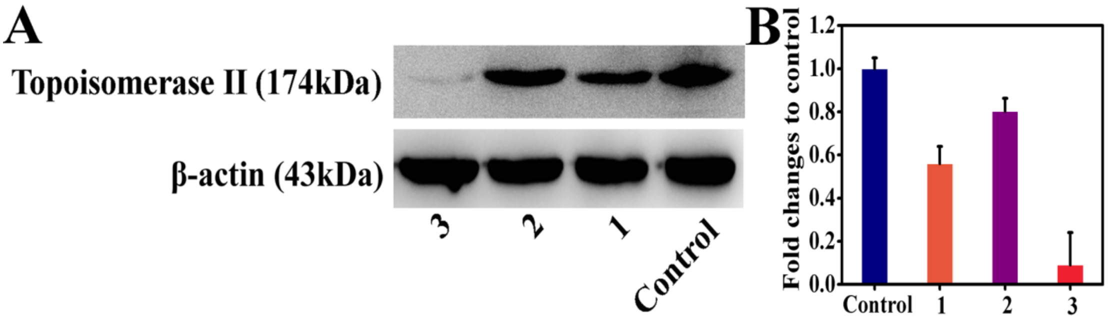

Figure S10. The protein levels of Topoisomerase II in HepG2 cells after treatment with complexes **1–3** (0.5 μM), respectively. (A) Topoisomerase II protein regulators protein levels in HepG2 cells were analyzed by western blot. (B) The whole-cell extracts were prepared and analyzed by Western blot analysis using antibodies against cell cycle protein regulators proteins. The same blots were stripped and reprobed with β-actin antibody to show equal protein loading. Western blotting bands from three independent measurements were quantified with Image J. in (B).

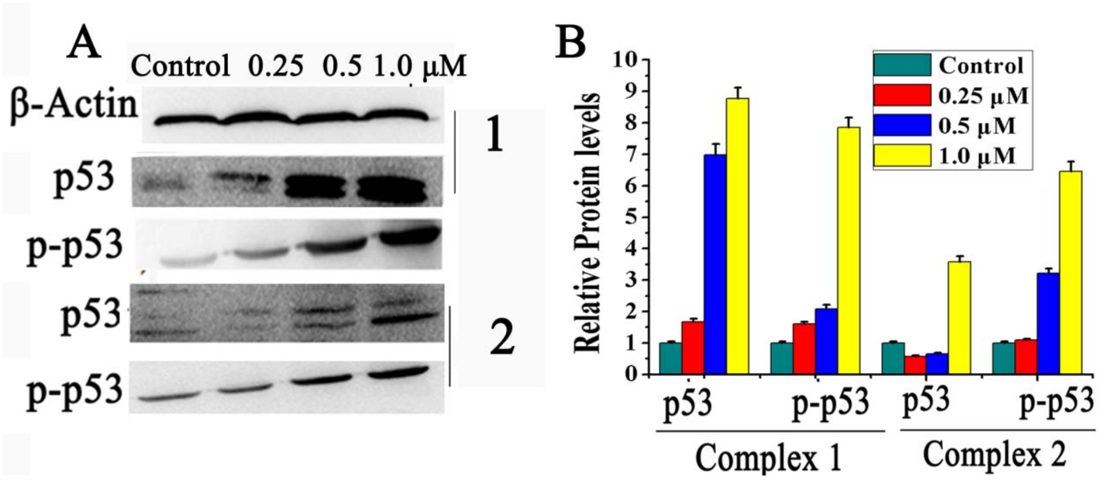

Figure S11.(a) Western blot analysis of p53 and p-p53 proteins in HepG2 cells with complex **1** and **2** at 0.25,0.5 and 1.0 μM for 24 h. (b) The relative expression of proteins from A, each band = (density of each band/density of β-actin band). Mean ± SD was from three independent measurements.

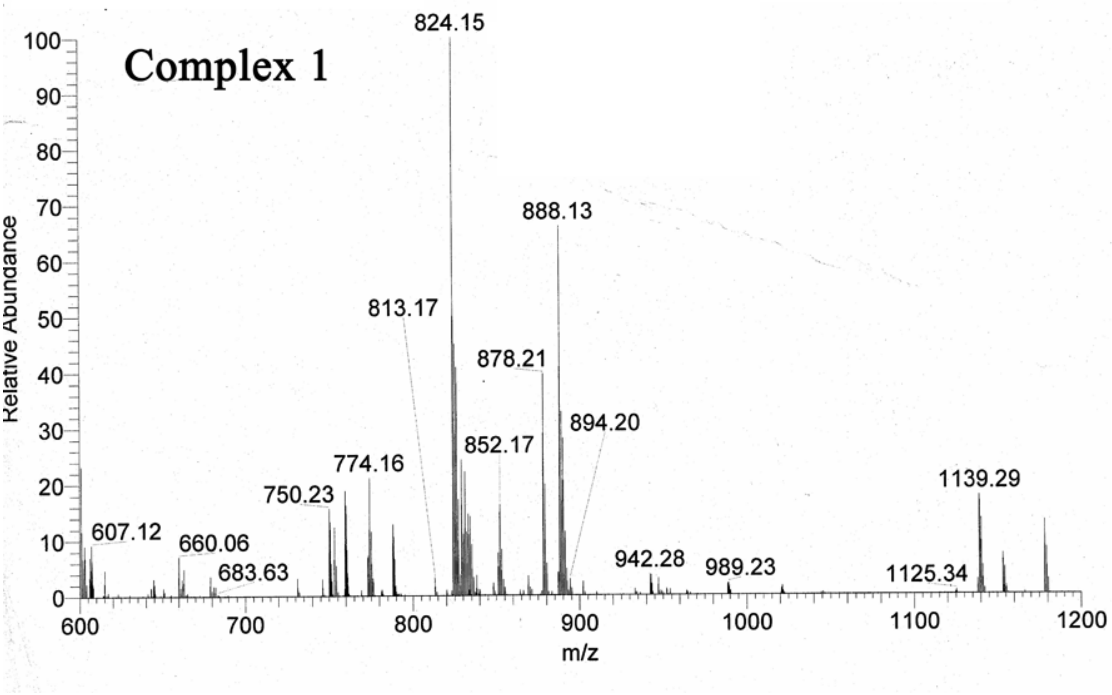

Figure S12 ESI-MS spectrum of complex **1** in MeOH / H_2O (1:500)

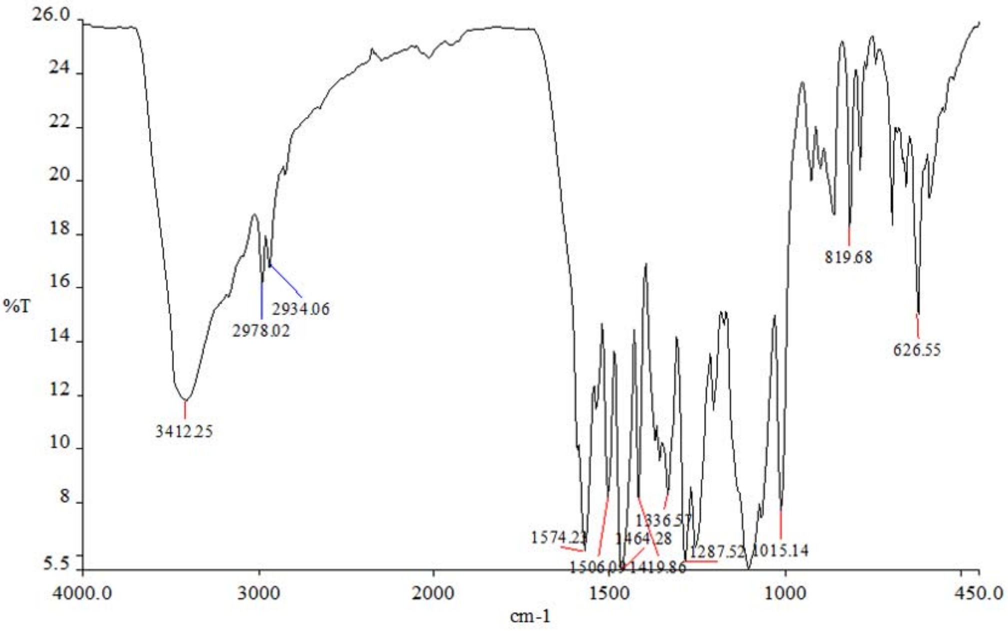

Figure S13. IR spectrum of complex **1.**

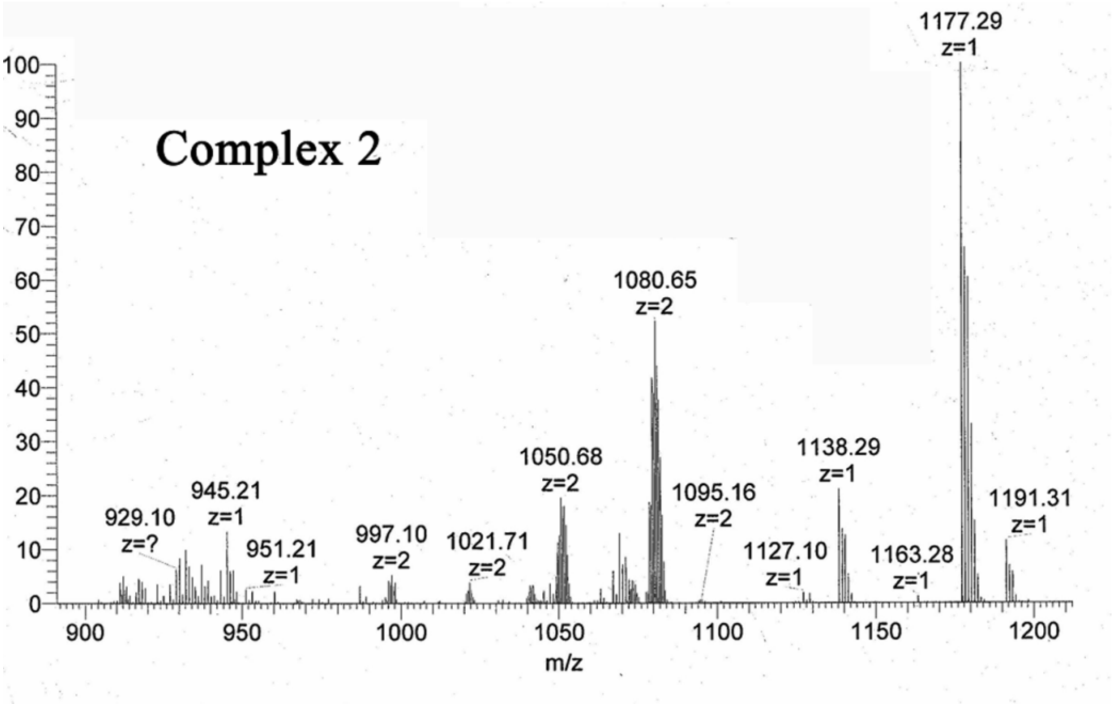

Figure S14. ESI-MS spectrum of complex **2** in MeOH / H_2O (1:500)

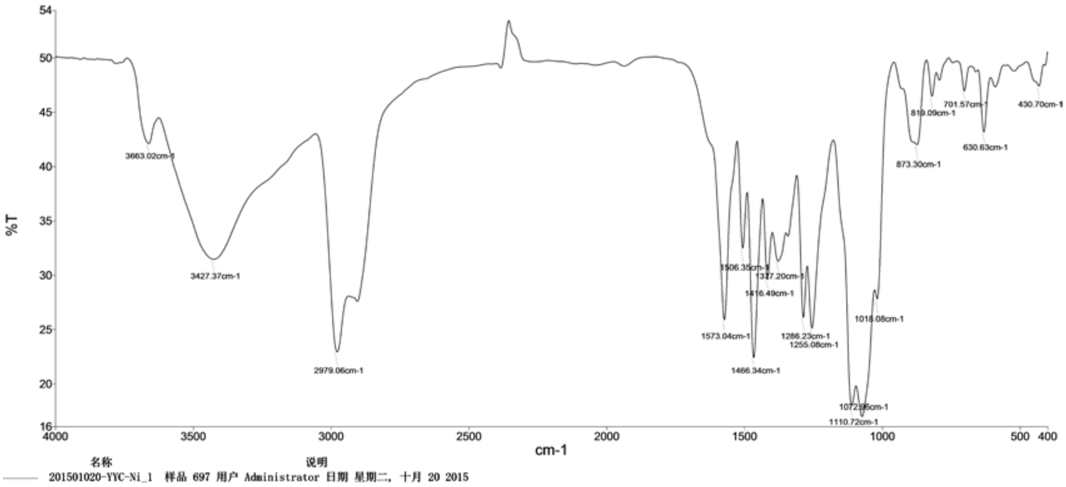

Figure S15.IR spectrum of complex **2.**

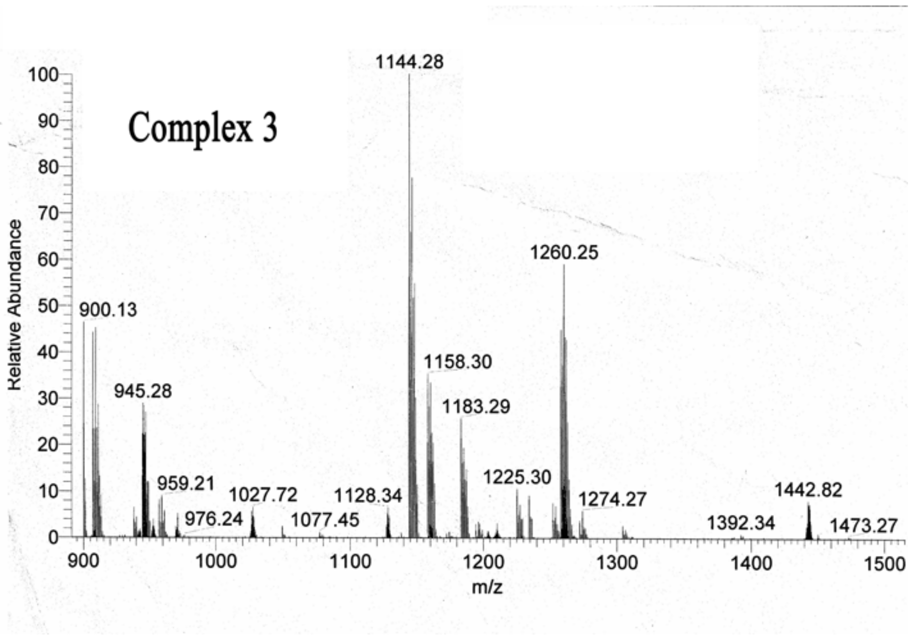

Figure S16. ESI-MS spectrum of complex **3** in MeOH / H_2O (1:500)

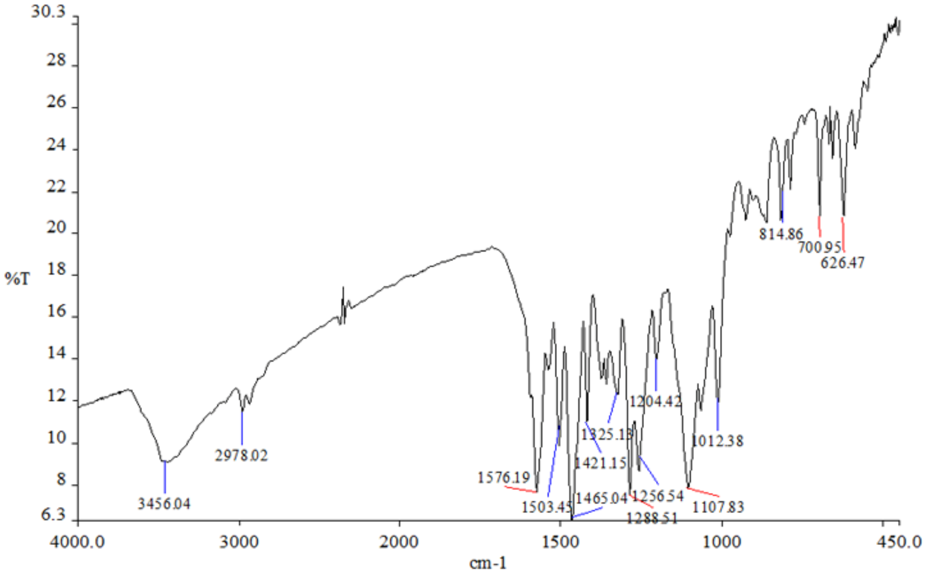

Figure S17.IR spectrum of complex **3.**
